# An Excipient-Free *Porphyra*/*Pyropia* sp.-Derived Polynucleotide Modulates Nitrative Stress in LPS-Stimulated Macrophages and Murine Acute Lung Injury

**DOI:** 10.3390/md24100361

**Published:** 2026-10-18

**Authors:** Sang-Seop Lee, Jisung Han, Won Se Lee, Su Jeong Ryu, Yung-Choon Yoo

**Affiliations:** 1 Department of Microbiology, College of Medicine, Konyang University, Daejeon 35365, Republic of Korea; wgd.aria@gmail.com; 2 All In One GENETECH Ltd., Busan 46508, Republic of Korea; aio@aiogenetech.com (J.H.); wslee8607@aiogenetech.com (W.S.L.); aiogenetech@gmail.com (S.J.R.); 3 DERMARE Co. Ltd., Busan 48480, Republic of Korea

**Keywords:** *Porphyra*/*Pyropia* sp., polydeoxyribonucleotide, marine polynucleotide, non-animal PDRN, selective radical scavenging, 3-nitrotyrosine, S-nitrosothiol, nitrative stress, acute lung injury, material characterization

## Abstract

Polydeoxyribonucleotide (PDRN) is a DNA-derived material conventionally sourced from salmon, which has prompted interest in scalable non-animal alternatives. Here, we characterized an excipient-free polynucleotide purified from cultivated *Porphyra*/*Pyropia* sp. (PS-PDRN) and examined its radical-scavenging properties and its effects on nitrative-stress-related readouts. Reference nuclear assemblies of three Bangiales species were uniformly GC-rich (65.08–71.26%), in contrast to 43.89% for *Oncorhynchus keta*, the source species of conventional PDRN, which places the algal origin of the material in genomic context. PS-PDRN showed negligible DPPH radical scavenging but dose-dependent ABTS radical-cation scavenging, a profile shared with salmon-derived PDRN and distinct from the pre-purification intermediate. In LPS-stimulated RAW 264.7 macrophages, 50 µg/mL PS-PDRN raised nitrite to 133% and lowered nitrate to 85% of the LPS group, increasing the nitrite-to-nitrate partitioning ratio, and lowered 3-nitrotyrosine to 77% while iNOS protein (119%) and the total S-nitrosothiol pool (97%) were maintained and NADPH oxidase activity was unchanged. In murine LPS-induced acute lung injury, PS-PDRN lowered lung 3-nitrotyrosine to 53% of the LPS group, whereas lung iNOS protein remained at 95% and *Nos2* at 67%, *Hmox1* rose to 148%, and total SOD activity exceeded the LPS level. These preliminary findings show that the purified *Porphyra*/*Pyropia* sp.-derived polynucleotide has a radical-scavenging profile distinct from its pre-purification material and lowers protein nitration without proportionate suppression of iNOS-related readouts. They support further evaluation of cultivated *Porphyra*/*Pyropia* sp. as a non-animal source of PDRN-class polynucleotides and provide a basis for mechanistic, manufacturing, and translational validation.

## 1. Introduction

Acute lung injury (ALI) and its severe form, acute respiratory distress syndrome, remain associated with substantial mortality and limited pharmacological options [1,2]. Lipopolysaccharide (LPS)-driven activation of alveolar macrophages initiates a cascade in which inducible nitric oxide synthase (iNOS/NOS2) and NADPH oxidase 2 (NOX2/CYBB) are co-induced. Nitric oxide (NO) and superoxide generated in parallel react at near-diffusion-limited rates to form peroxynitrite, which nitrates protein tyrosine residues and contributes to tissue injury [3]. Because of this chemistry, iNOS has long been treated as a target for suppression. That framing has become harder to sustain. In cutaneous wound repair, endotoxin tolerance, autoimmune inflammation, and inflammatory bowel disease, iNOS expression remains high while inflammatory output falls, and genetic or pharmacological loss of iNOS worsens rather than improves outcome [4,5,6,7,8].

Polydeoxyribonucleotide (PDRN), an adenosine A_2A_ receptor agonist used clinically for tissue repair, shows the same pattern: iNOS and NO metabolites rise while TNF-α, IL-1β, and IL-6 fall [9,10,11]. These observations suggest that the consequences of iNOS induction depend on the redox environment in which NO is produced rather than on the amount of NO alone [12]. NADPH oxidase-derived oxidant input is a principal determinant of that environment, and NOX-NOS crosstalk has been proposed as a general axis linking oxidant load to nitrosative and nitrative outcomes [13]; a marine algal extract acting on this axis attenuated NLRP3 inflammasome activation in murine colitis [14], and marine algal extracts have been evaluated in a related intranasal LPS-induced murine acute lung injury model [15]. PDRN is currently obtained predominantly from salmonid sources [11]. Cultivated red algae of the genus *Pyropia* are an attractive non-animal source: they are farmed at scale, have a long history of dietary use, and contain nucleic acids in recoverable quantity [16,17].

*Pyropia*-derived preparations have been reported to exert anti-inflammatory effects in macrophages and in murine ALI [18,19]. However, the extraction formulations used in those preparations contained green tea extract and tocopherol, introducing antioxidant co-components that complicated attribution of radical-scavenging activity to the polynucleotide itself [18,19]. The macrophage study also measured NO output as nitrite alone [18], which leaves the distribution of NO among nitrite, nitrate, S-nitrosothiols, and protein nitration unresolved. Whether a purified, excipient-free preparation retains such activity, and by what mechanism, has not been established. Here we characterize an excipient-free *Porphyra*/*Pyropia* sp.-derived polynucleotide (PS-PDRN). We first ask whether its radical-scavenging profile differs from that of a conventional antioxidant, from salmon-derived PDRN, and from the pre-purification intermediate.

Acute lung injury was selected as the in vivo setting because it places the nitrative readouts of interest in a tissue where they are well characterized. LPS-induced lung injury co-induces iNOS and NOX2 in alveolar macrophages and infiltrating neutrophils, and the resulting peroxynitrite chemistry leaves a measurable 3-nitrotyrosine footprint in lung tissue [3,6]. The intranasal LPS model has also been applied to a related *Porphyra*-derived polynucleotide [19] and to other marine algal extracts [15], so the present data can be read against earlier results from the same system. Translationally, the findings bear on acute respiratory distress syndrome, for which pharmacological options remain limited [1,2].

We then examine, in LPS-stimulated macrophages and in murine ALI, how NO output, protein nitration, NADPH oxidase, and antioxidant response genes respond. Experimental measurements form the core of the study; each set of observations is examined against a complementary source of context—genome-informed characterization for material identity, a structured survey of the iNOS literature for the directionality of NO-related readouts, and pharmacological comparators for mechanistic positioning. The study is framed as an early-phase scoping analysis intended to delineate plausible mechanistic directions for subsequent hypothesis-driven testing rather than to establish a mechanism.

## 2. Results

### 2.1. Physical Characterization and Source-Genome Context of PS-PDRN

**Figure 1:**
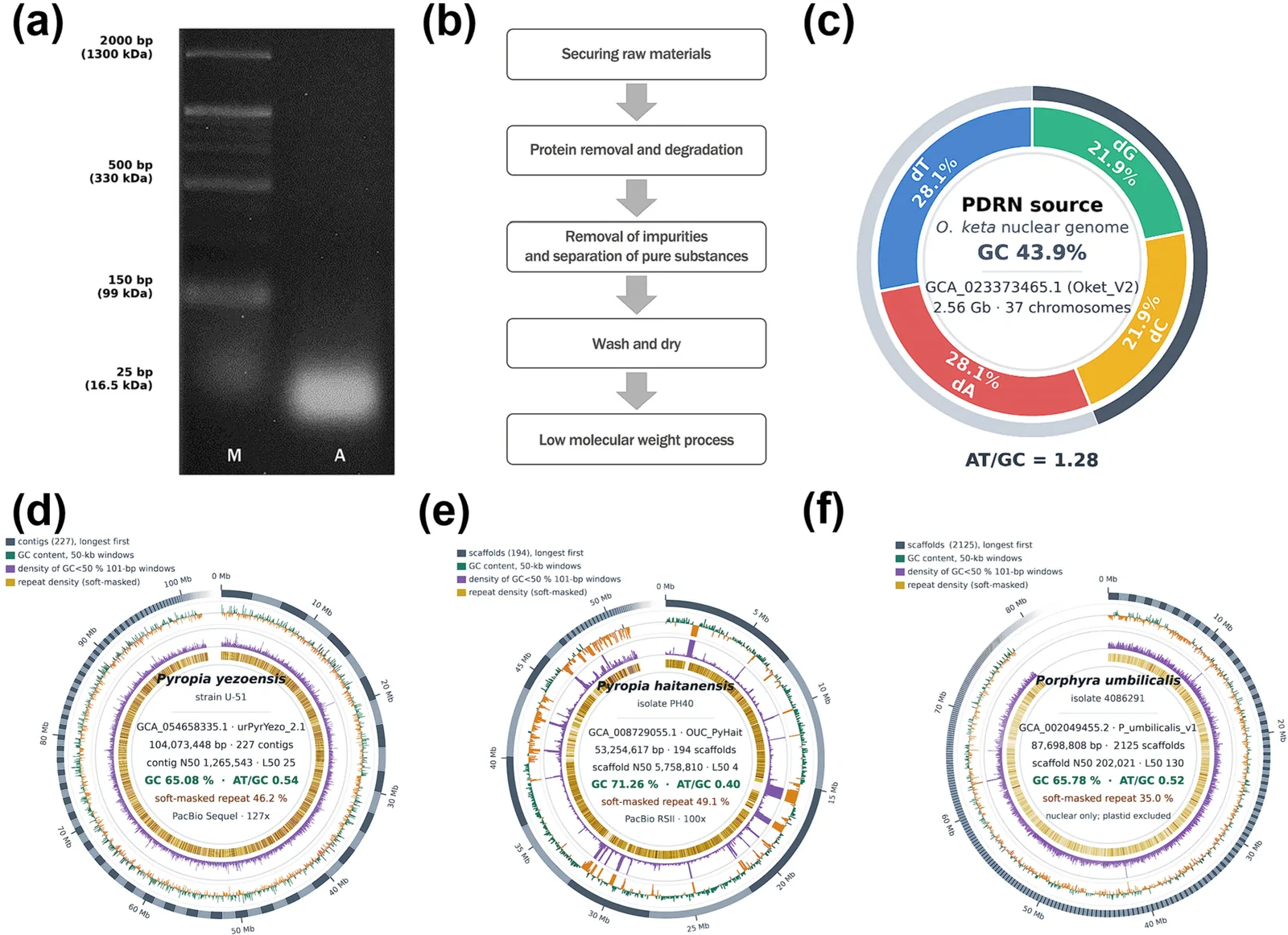
Physical characterization, manufacturing workflow, and reference genomic context for PS-PDRN. (**a**) Agarose gel electrophoresis of the PS-PDRN preparation (lane A) alongside a molecular size marker (lane M); the nominal molecular weight corresponding to each marker band is given in parentheses. The gel was run on 1.5% agarose by the manufacturer on the lot used in this study; the product migrates as a single diffuse band ahead of the smallest (25 bp) marker band, and the uncropped gel is shown in Figure S3. (**b**) Manufacturing workflow, from raw material sourcing through enzymatic protein removal and degradation, removal of impurities and separation of the purified substance, washing and drying, to the final low-molecular-weight processing step. (**c**) Nucleotide composition of the nuclear genome of *Oncorhynchus keta* (chum salmon), the source species of conventional PDRN, shown as reference context for the comparator used in this study. The inner ring gives the mole fraction of each deoxynucleotide, assuming Chargaff base pairing; the outer ring gives the GC (dark) and AT (light) fractions. (**d**–**f**) Circos representations of three published Bangiales (red algal) reference assemblies: (**d**) *Pyropia yezoensis* strain U-51 (GCA_054658335.1, urPyrYezo_2.1), (**e**) *Pyropia haitanensis* isolate PH40 (GCA_008729055.1, OUC_PyHait), and (**f**) *Porphyra umbilicalis* isolate 4086291 (GCA_002049455.2, P_umbilicalis_v1; nuclear sequences only, the plastid genome excluded). In each panel, rings from outermost to innermost show: the contigs or scaffolds ordered by decreasing length, with a coordinate scale in Mb; GC content in 50 kb windows plotted as deviation from that genome’s own mean (green above, orange below; ±12 percentage points full scale); the proportion of short (101 bp) windows with GC below 50% within each 50 kb window; and soft-masked repeat density. Assembly statistics are summarized at the centre of each panel. All GC values were calculated directly from assembly base counts. Across the three red algal genomes GC content ranges from 65.08% to 71.26%, compared with 43.89% for *O. keta*.

The purified material migrated as a single diffuse band ahead of the smallest (25 bp) marker band on 1.5% agarose gel electrophoresis (Figure 1a; uncropped gel in Figure S3), consistent with the controlled molecular-weight reduction step of the manufacturing workflow (Figure 1b). These conditions identify the material as a low-molecular-weight preparation, and a precise fragment-size estimate would require a higher-resolution separation.

To place the material in taxonomic and compositional context, we examined publicly available reference assemblies. Three Bangiales nuclear assemblies—*Pyropia yezoensis* (syn. *Neopyropia yezoensis* [16]) U-51 (GCA_054658335.1; 104.07 Mb, 227 contigs), *Pyropia haitanensis* PH40 (GCA_008729055.1; 53.25 Mb, 194 scaffolds), and *Porphyra umbilicalis* (GCA_002049455.2; 87.70 Mb, 2125 scaffolds)—are uniformly GC-rich, with GC contents of 65.08%, 71.26%, and 65.78%, respectively (Figure 1d–f). By contrast, the nuclear assembly of *Oncorhynchus keta* (GCA_023373465.1, Oket_V2), the source species of conventional animal-derived PDRN, has a GC content of 43.89% (Figure 1c). Red algal and salmonid source genomes are therefore separated by more than 20 percentage points in GC content, irrespective of which Bangiales species is taken as the reference. These assemblies are presented as source-level context and do not constitute compositional measurements of PS-PDRN; the raw material is designated *Porphyra*/*Pyropia* sp.

Purification markedly reduced non-nucleic-acid constituents: total polyphenol content decreased from 21.7 mg gallic acid equivalents (GAE)/g dry weight in the pre-purification intermediate to 1.0 ± 0.073 mg GAE/g dry weight in PS-PDRN. The A_260_/A_280_ ratio of PS-PDRN was 1.93 ± 0.06, consistent with the value of 1.89 recorded in the manufacturer’s quality control for this lot, and the A_260_/A_230_ ratio was 1.91 ± 0.04; the salmon-derived reference gave 1.82 ± 0.02 and 2.09 ± 0.01. Residual protein and polysaccharide were 0.60 ± 0.11% and 1.83 ± 0.14% of DNA content, within the predefined quality-control limits, and endotoxin was below 0.1 EU/mg.

### 2.2. PS-PDRN Scavenges ABTS but Not DPPH Radicals

**Figure 2:**
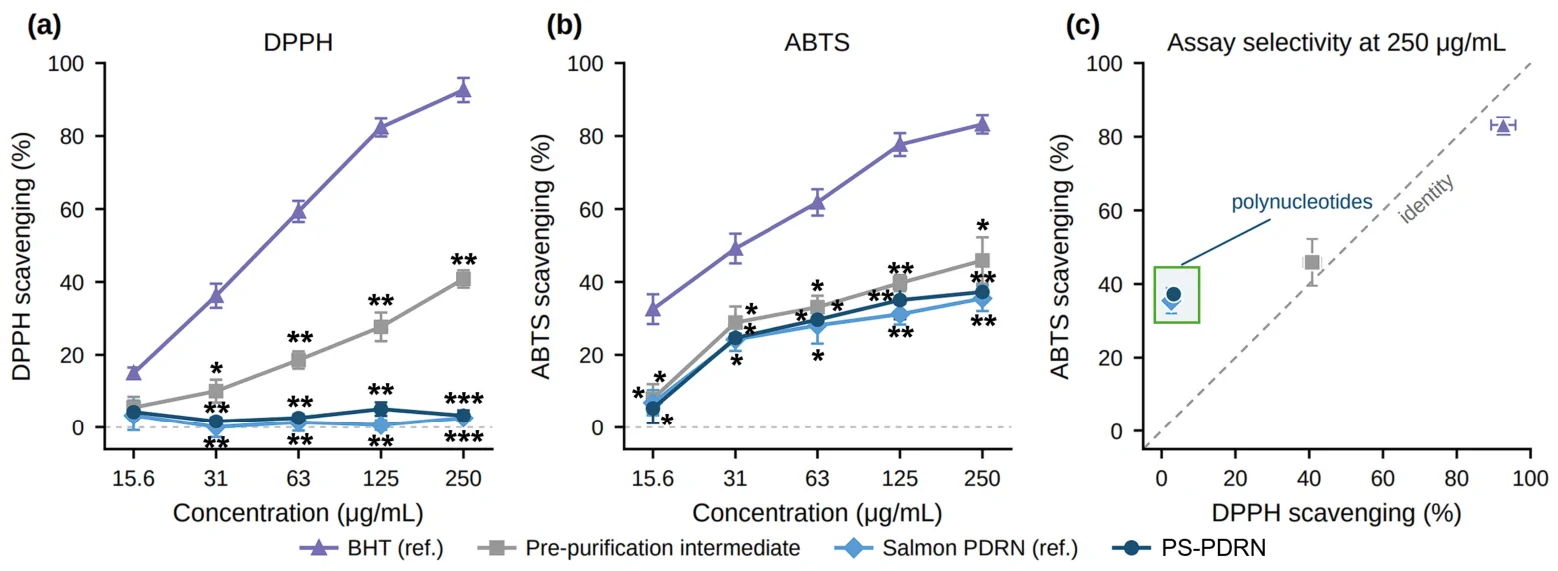
Selective radical scavenging by PS-PDRN. (**a**) DPPH and (**b**) ABTS radical-scavenging activity over 15.6–250 µg/mL for PS-PDRN, salmon-derived PDRN, the pre-purification intermediate, and butylated hydroxytoluene (BHT); the horizontal dashed lines indicate the baseline (0% scavenging activity). (**c**) DPPH plotted against ABTS scavenging at 250 µg/mL; the dashed line indicates equal activity in both assays and the boxed region highlights the two purified polynucleotide preparations. Data are mean ± SD of *n* = 3 independent experiments. Where shown, *, **, and *** denote *p* < 0.05, *p* < 0.01, and *p* < 0.001, respectively, versus the concentration-matched BHT reference.

The two assays gave sharply different results (Figure 2a,b). Butylated hydroxytoluene scavenged both radicals in a concentration-dependent manner, reaching 92.6% (DPPH) and 83.2% (ABTS) at 250 µg/mL. The pre-purification intermediate also scavenged both, reaching 40.8% and 45.9%. PS-PDRN, in contrast, showed minimal DPPH activity across the entire range tested (≤4% at 250 µg/mL) while scavenging 37.2% of ABTS radicals at the same concentration. Salmon-derived PDRN behaved almost identically (2.6% and 35.5%).

Plotting the two assays against each other at 250 µg/mL separates the preparations clearly (Figure 2c). Butylated hydroxytoluene and the pre-purification intermediate lie close to the identity line, indicating comparable activity in both assays. The two polynucleotides fall far above it, occupying an overlapping region defined by substantial ABTS and negligible DPPH scavenging.

Two points follow. First, the scavenging profile of PS-PDRN is not distinguishable from that of salmon-derived PDRN, supporting the closely similar radical-scavenging profile of the two preparations. Second, the pre-purification intermediate retains DPPH activity that PS-PDRN does not, consistent with removal during purification of constituents capable of hydrogen-atom transfer.

### 2.3. PS-PDRN Raises the Nitrite Fraction of NO Output and Lowers Nitration While iNOS Protein Is Maintained

**Figure 3:**
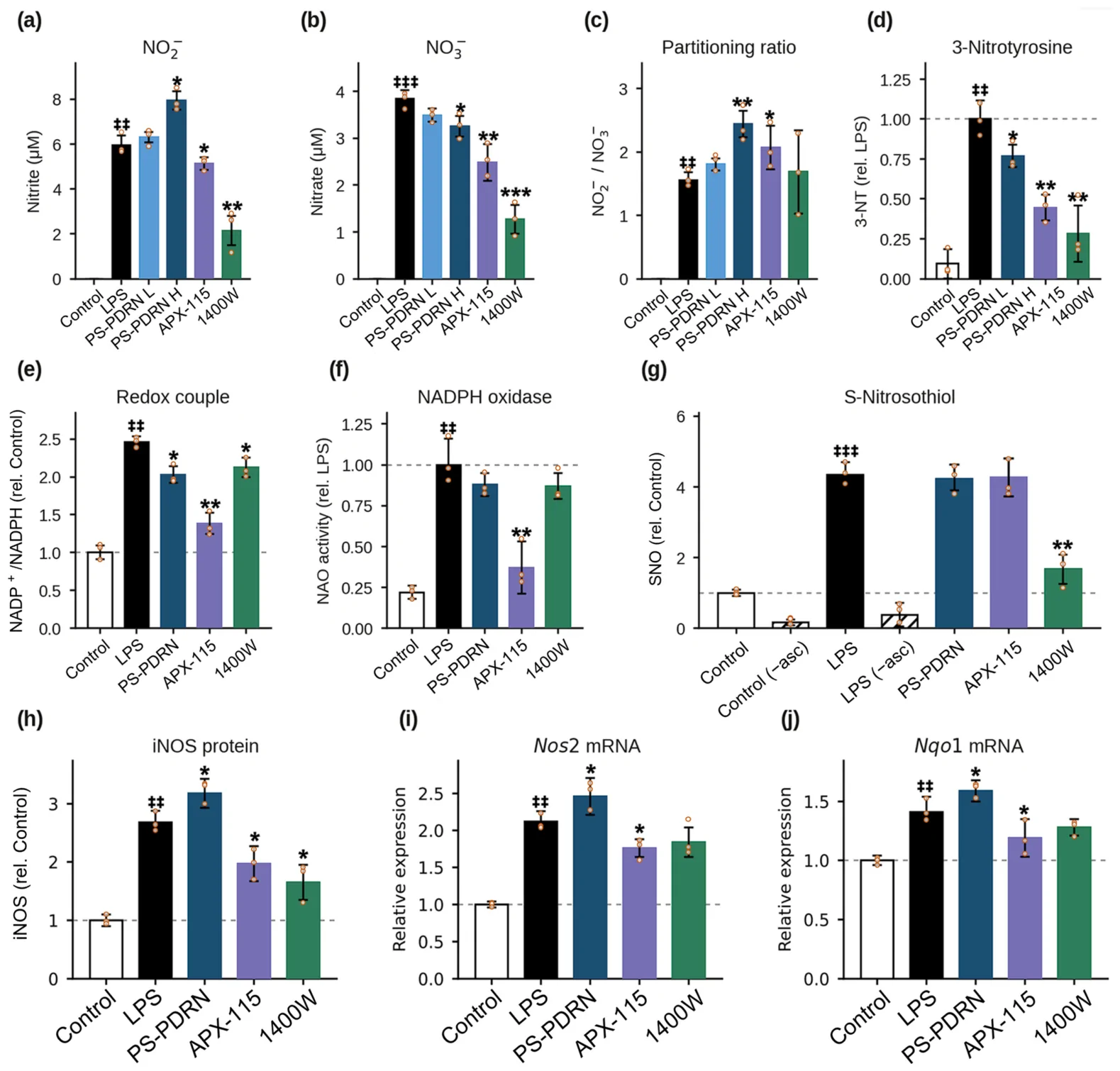
Macrophage NO output, NO fate, and redox status in LPS-stimulated RAW 264.7 cells. (**a**) Nitrite, (**b**) nitrate, and (**c**) the NO_2_^−^/NO_3_^−^ partitioning ratio in culture supernatant; (**d**) 3-nitrotyrosine relative to the LPS group; (**e**) NADP^+^/NADPH relative to untreated control cells; (**f**) NADPH oxidase (NAO) activity relative to the LPS group; (**g**) total S-nitrosothiol relative to untreated control cells, hatched bars showing the ascorbate-omitted wells processed in parallel as an assay-specificity control; (**h**) iNOS protein relative to untreated control cells; (**i**) *Nos2* and (**j**) *Nqo1* transcript levels, normalized to *Gapdh* and *Actb* and expressed relative to untreated control cells. PS-PDRN was applied at 25 (L) and 50 (H) µg/mL in (**a**–**c**) and at 50 µg/mL in (**d**–**j**); APX-115 (5 µM) and 1400W (10 µM) were applied on the same co-treatment schedule. The horizontal dashed lines across the subfigures indicate a value of 1, representing the baseline for the respective reference group. Data are mean ± SD of *n* = 3 independent experiments, with individual experiments shown as points. ‡‡, and ‡‡‡ denote *p* < 0.05, *p* < 0.01, and *p* < 0.001 versus untreated control, respectively; *, **, and *** denote *p* < 0.05, *p* < 0.01, and *p* < 0.001 versus the LPS group, respectively.

RAW 264.7 viability, measured by WST-8 reduction after 24 h, remained above 80% of the corresponding control under every condition used in the functional experiments (Figure S4). With 25 and 50 µg/mL PS-PDRN it was 96.9% and 91.0% of untreated cells without LPS and 89.8% and 84.3% of the LPS group with LPS, and APX-115 and 1400W gave 87.5% and 88.4% with LPS. LPS alone reduced the WST-8 signal to 78.6% of untreated cells. The 84.3% value at 50 µg/mL therefore represents an approximately 16% decrease relative to LPS alone. Viability fell to approximately 75% of the corresponding control at 100 µg/mL, a concentration not used in the functional experiments. In LPS-stimulated RAW 264.7 macrophages, PS-PDRN increased nitrite in a concentration-dependent manner, reaching 7.94 µM at 50 µg/mL versus 5.95 µM in the LPS group (133% of LPS; Figure 3a). Nitrate moved in the opposite direction, falling to 3.25 µM from 3.84 µM (85%; Figure 3b), so that the NO_2_^−^/NO_3_^−^ ratio rose from 1.55 to 2.44 (Figure 3c). Total NO metabolites (nitrite plus nitrate), the integrated product of NO synthesis over the culture period, were 114% of the LPS group (11.19 versus 9.79 µM). Both pharmacological comparators reduced nitrite and nitrate together: APX-115, a pan-NADPH oxidase inhibitor [20], to 86% and 65%, and 1400W, a selective iNOS inhibitor [21], to 36% and 33% respectively. Without LPS, PS-PDRN raised nitrite to 0.52 ± 0.03 and 1.97 ± 0.27 µM at 25 and 50 µg/mL and nitrate to 0.09 ± 0.05 and 0.17 ± 0.03 µM, against values at the detection limit in untreated cells (Figure S4b,c), so the material supports a low level of NO production on its own, about a third of the LPS-stimulated nitrite output at the higher concentration.

3-Nitrotyrosine fell to 77% of the LPS group (Figure 3d), while the NADP^+^/NADPH ratio decreased from the LPS-elevated state toward the control level (Figure 3e). NADPH oxidase activity remained at 88% of the LPS group and was not significantly different from LPS alone, whereas APX-115 reduced activity to approximately 37% (Figure 3f). The iNOS inhibitor 1400W strongly reduced both nitrite and nitrate while leaving NADPH oxidase activity near the LPS level. Conversely, APX-115 reduced nitration and nitrate together with direct NADPH oxidase inhibition. These pharmacological profiles distinguish suppression of NO synthesis from suppression of oxidant input, the two arms of the NOX-NOS axis [13], and provide mechanistic context for the PS-PDRN phenotype without establishing a single causal route.

Neither iNOS protein nor *Nos2* transcript decreased under PS-PDRN. Both were higher than in the LPS group (119% and 116%, respectively; Figure 3h,i), so the fall in 3-nitrotyrosine occurred without a reduction in iNOS protein abundance. Because protein abundance is an indirect index of catalytic activity, the total NO metabolite output described above provides the more direct indication that NO synthesis was maintained. The two comparators separated along the same axis: values were modestly lower with APX-115 (74% and 83%) and with 1400W (67% and 86%). *Nqo1* transcript was likewise higher with PS-PDRN than with LPS alone (113%; Figure 3j), indicating that an antioxidant response was engaged under co-treatment as well as in vivo. *Nqo1*, *Hmox1* and SOD are established Nrf2 target genes co-regulated as an antioxidant programme in lung macrophages [22].

Total S-nitrosothiol rose 4.3-fold above untreated control cells with LPS and was unchanged by PS-PDRN (97% of the LPS group) or APX-115 (108%), whereas 1400W reduced it to 38% (Figure 3g). Wells carried through the same procedure with ascorbate omitted from the reduction step retained 16% and 9% of the corresponding control and LPS signal, confirming that the measurement was ascorbate-dependent. The S-nitrosothiol pool therefore followed NO synthesis rather than the nitration readout: under PS-PDRN it was maintained at the LPS level while 3-nitrotyrosine fell to 77%, and it was lost only when NO synthesis itself was inhibited.

Taken together, PS-PDRN differed from both pharmacological comparators. At the higher concentration it increased nitrite while lowering nitrate and 3-nitrotyrosine; it maintained the S-nitrosothiol pool at the LPS level; iNOS protein and *Nos2* transcript were higher, not lower, than in the LPS group; and NADPH oxidase activity was not significantly inhibited. This combination is consistent with altered partitioning of NO-derived products rather than with a reduction in NO generation. Nitrite, total NO metabolites, and iNOS protein rose under PS-PDRN whereas both comparators lowered NO output, indicating that the PS-PDRN-treated cultures retained their capacity to produce NO. However, the approximately 16% reduction in WST-8 signal at 50 µg/mL relative to LPS alone may reflect changes in cell number or metabolic activity and could have indirectly influenced the functional readouts. This possibility cannot be excluded by the present viability assay.

### 2.4. The Observed Pattern Recurs Across the iNOS Literature

**Figure 4:**
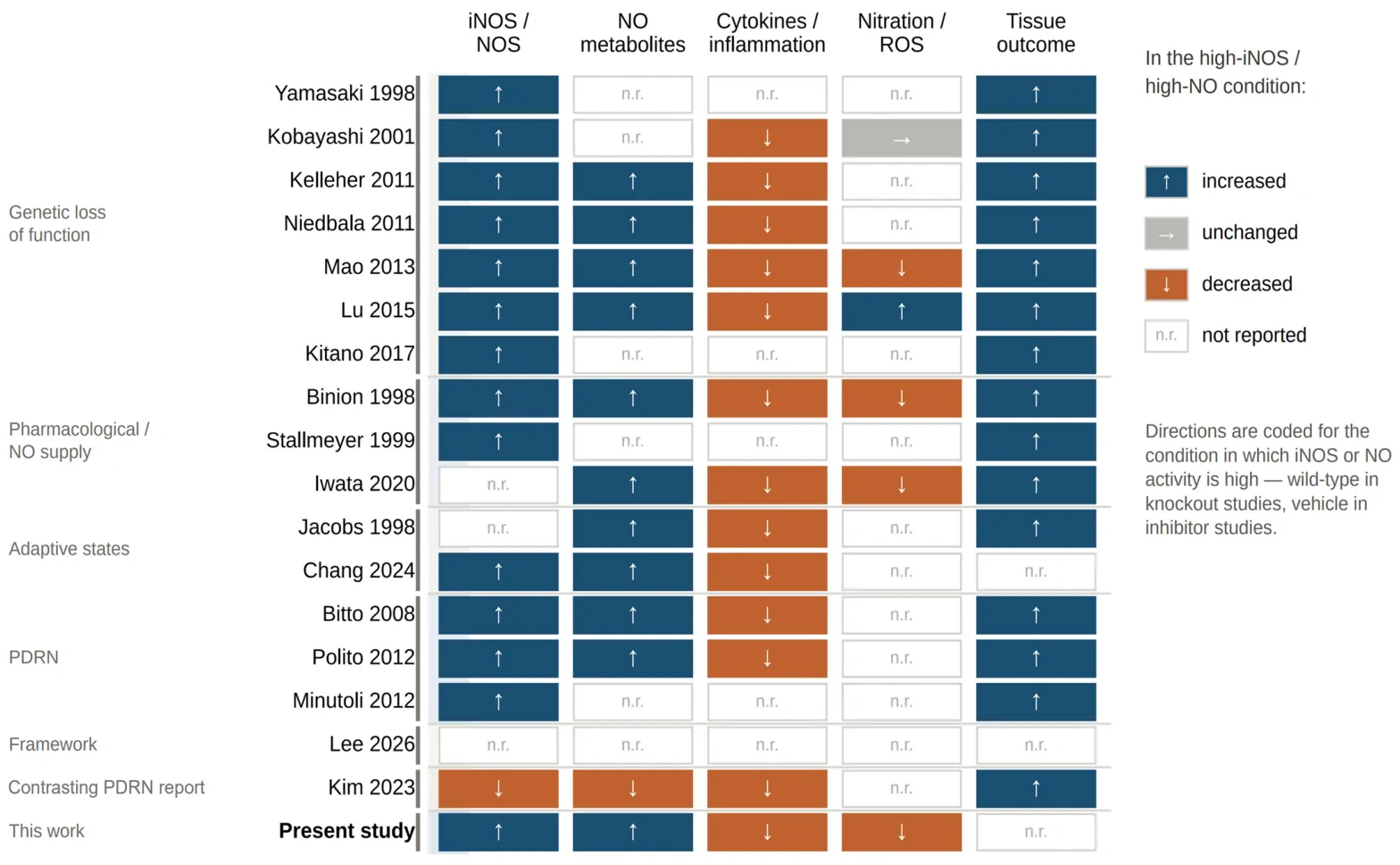
Directional map of reported iNOS/NO responses and concurrent inflammatory, nitrative, and outcome measures. Rows are grouped by evidence class rather than chronology. All directions are coded for the condition in which iNOS or NO activity is high—wild-type in knockout studies, vehicle in inhibitor studies. Entries are: genetic loss of function, Yamasaki 1998 [5], Kelleher 2011 [6], Mao 2013 [7], Lu 2015 [8], Kobayashi 2001 [24], Niedbala 2011 [25], and Kitano 2017 [26]; pharmacological inhibition or NO supply, Binion 1998 [4], Stallmeyer 1999 [27], and Iwata 2020 [28]; adaptive states, Jacobs 1998 [29] and Chang 2024 [30]; PDRN, Bitto 2008 [9], Polito 2012 [10], and Minutoli 2012 [23]; framework, Lee 2026 [12]; and contrasting PDRN report, Kim 2023 [18]. Lee 2026 [12] is a hypothesis article containing no primary data and is shown without coded entries. Kim 2023 [18] is presented separately as a contrasting PDRN report and is compared with the present study in Table 1. This survey is descriptive and was not conducted as a systematic review; studies differ in model, preparation, and readout, and tabulated directions are not weighted by effect magnitude. Full details are given in Table S1.

The structured survey retrieved primary studies reporting both NOS/NO status and inflammatory, nitrative, or outcome measures (Figure 4; Table S1). Across genetic loss-of-function, pharmacological, and adaptive-state studies, the high-iNOS condition was associated with reduced inflammatory output and improved or unchanged tissue outcome in every entry. Three PDRN-specific reports showed the same direction [9,10,23].

Two features of the survey bear on the present work. First, nitration or ROS were reported in the retrieved studies; where measured, they were unchanged [24] or reduced [4,7,28], with the exception of Lu et al., in which tyrosine nitration of IRF5 constitutes the inhibitory signal rather than an injury marker [8]. Second, a single retrieved study reported the opposite direction for a *Pyropia*-derived preparation [18]; it differed in preparation purity, treatment schedule, and NO readout, and is compared with the present study in Section 3.1 and Table 1.

### 2.5. PS-PDRN Lowers Lung 3-Nitrotyrosine Relative to Total NO Metabolite Output

LPS challenge produced the expected features of acute lung injury: body temperature rose from 34.6 ± 0.21 to 38.2 ± 0.70 °C and the lung wet/dry ratio from 6.4 to 10.3. Both were attenuated by dexamethasone, a corticosteroid comparator used to limit neutrophilic inflammation in murine acute lung injury [31] (36.2 °C; 7.2) and, to a lesser extent, by PS-PDRN (37.0 °C; 8.6) (Figure S1). Serum TNF-α, IL-1β, and CCL5 were also increased by LPS and attenuated by treatment, with the overall suppression being more pronounced with dexamethasone than with PS-PDRN (Figure S2).

**Figure 5:**
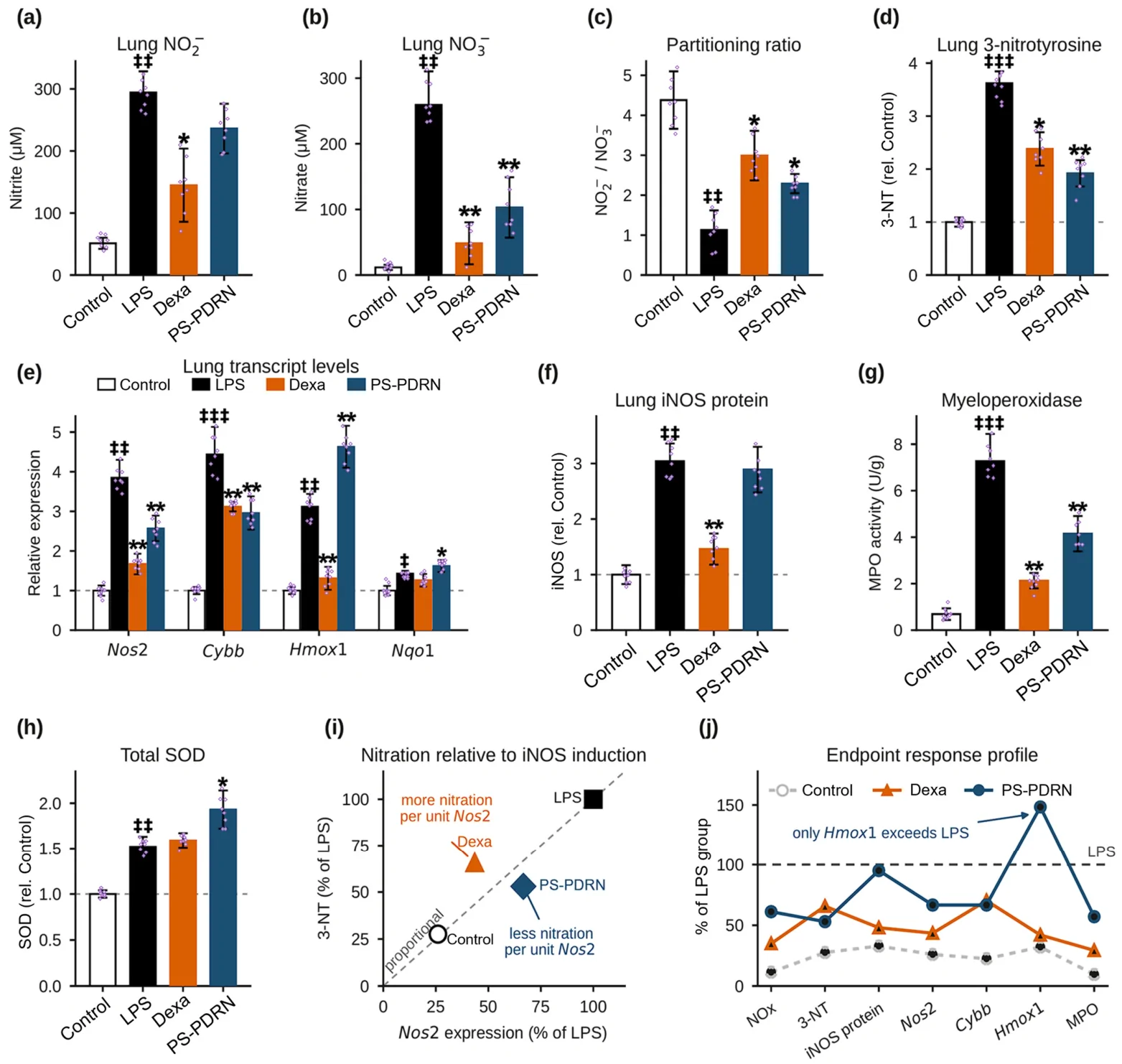
Redox and nitrative status in LPS-induced murine acute lung injury. (**a**) Lung nitrite, (**b**) nitrate, and (**c**) their partitioning ratio; (**d**) lung 3-nitrotyrosine relative to controls; (**e**) lung transcript levels of *Nos2*, *Cybb*, *Hmox1*, and *Nqo1*, normalized to *Gapdh* and *Actb* and expressed relative to controls; (**f**) lung iNOS protein relative to controls; (**g**) MPO activity; and (**h**) total SOD activity relative to controls. (**i**) 3-Nitrotyrosine plotted against *Nos2* expression, both as a percentage of the LPS group; the dashed line is the proportional-reference line (y = x), not a fitted regression. (**j**) Selected endpoint response profile expressed as a percentage of the LPS group. In (**d**–**f**,**h**), the horizontal dashed line at 1 marks the untreated control level, to which these values are normalized. Data are mean ± SD of *n* = 8 animals per group, with individual animals shown as points. ‡, ‡‡, and ‡‡‡ denote *p* < 0.05, *p* < 0.01, and *p* < 0.001 versus untreated control, respectively; *, and ** denote *p* < 0.05, and *p* < 0.01 versus the LPS model control, respectively.

Lung nitrite and nitrate were both elevated by LPS (Figure 5a,b), and the NO_2_^−^/NO_3_^−^ ratio fell from 4.38 in control lungs to 1.13 (Figure 5c). PS-PDRN partially restored the ratio to 2.29, as did dexamethasone (2.99). Total lung NO metabolites were 61% of the LPS group in PS-PDRN-treated animals and 35% with dexamethasone. In BALF, by contrast, total NO metabolites in PS-PDRN-treated animals were 102% of the LPS group; the two compartments are reported separately and are not combined. Lung 3-nitrotyrosine rose 3.6-fold with LPS and was reduced to 53% of the LPS group by PS-PDRN and to 66% by dexamethasone (Figure 5d). Expressed per unit of total lung NO metabolites, 3-nitrotyrosine was therefore 0.87 times the LPS value under PS-PDRN and 1.89 times under dexamethasone.

Transcript analysis showed that the two treatments achieved these reductions differently (Figure 5e). Dexamethasone reduced *Nos2* to 44% of the LPS group, whereas PS-PDRN left it at 67%. *Cybb* was reduced comparably by both (70% and 67%). *Hmox1*, induced 3.1-fold by LPS alone, was further elevated to 148% of the LPS group by PS-PDRN but reduced to 42% by dexamethasone; *Nqo1* followed the same direction (114% and 88%).

The two treatments separated further at the protein level (Figure 5f). Lung iNOS protein rose 3.0-fold with LPS; dexamethasone reduced it to 48% of the LPS group, whereas under PS-PDRN it remained at 95% and did not differ significantly from LPS alone. The partial reduction in *Nos2* transcript under PS-PDRN was therefore not accompanied by a corresponding fall in enzyme protein, over the same comparison in which 3-nitrotyrosine fell to 53%.

MPO activity was reduced by both treatments, more markedly by dexamethasone (29% of LPS) than by PS-PDRN (57%; Figure 5g). Total SOD activity, elevated 1.5-fold by LPS alone, was 1.9-fold in PS-PDRN-treated lungs but unchanged relative to LPS by dexamethasone (1.6-fold) (Figure 5h).

Plotting 3-nitrotyrosine against *Nos2* expression separated the two treatments (Figure 5i). The dashed y = x line denotes proportional change and is not a fitted regression. Dexamethasone fell above this reference, indicating more residual nitration per unit of *Nos2* expression, whereas PS-PDRN fell below it. Among the selected endpoints displayed in Figure 5j, *Hmox1* was the only measure for which PS-PDRN exceeded the LPS group.

## 3. Discussion

### 3.1. Relation to Earlier Porphyra/Pyropia-Derived Preparations

Two earlier studies examined *Porphyra*-derived polynucleotide preparations in the experimental systems used here: Kim et al. in LPS-stimulated RAW 264.7 macrophages [18] and Lee et al. in intranasal LPS-induced murine acute lung injury [19]. Table 1 compares these studies with the present work in material composition, design, readouts, and principal findings. Three differences define what the present study adds. The earlier preparations were produced with extraction formulations containing green tea extract and tocopherol, while PS-PDRN is excipient-free at the purified-product stage and its residual polyphenol content was quantified (Section 2.1). The earlier studies characterized the inflammatory response through nitrite, cytokines, and MAPK signalling; the present study resolves NO into nitrite, nitrate, S-nitrosothiol, and protein nitration and measures iNOS protein, NADPH oxidase activity, and Nrf2 target genes in the same samples. The macrophage study used a 12 h pre-treatment, and the present in vitro experiments used co-treatment. The studies agree on the direction of the inflammatory response and differ in NO output, and the remainder of this section and Section 3.3 consider how the differences in readout and schedule bear on that divergence.

The reduced nitrite accumulation reported for the earlier macrophage preparation [18] may appear discordant with the increase observed here. The two sets of measurements, however, address different aspects of NO biology. The Griess reaction quantifies nitrite, one of several terminal products of NO metabolism; the remainder partitions into nitrate, peroxynitrite-derived species, S-nitrosothiols, and protein-bound nitration products. A decrease in nitrite is compatible with either reduced NO synthesis or redirection of NO among these fates, and nitrite measurement alone cannot distinguish them.

By resolving nitrite, nitrate, and 3-nitrotyrosine in parallel, the present study addresses a level of NO chemistry not accessible through nitrite quantification. On this reading the earlier reports and the present observations describe different layers of the same process. This interpretation is consistent with the NOx:3-NT partitioning signature proposed previously as a readout of NO fate [12]. Direct measurement of the S-nitrosothiol pool supports this reading: under PS-PDRN the pool was maintained at the LPS level while 3-nitrotyrosine fell, and it was reduced only by 1400W, indicating that the maintained pool was NOS-derived rather than a residual background signal (Figure 3g).

### 3.2. Preparation Purity Determines the Scavenging Phenotype

The pre-purification intermediate scavenged DPPH radicals to 41% at 250 µg/mL, whereas PS-PDRN did not exceed 4% at the same concentration. Earlier related *Pyropia* preparations were produced with antioxidant-containing extraction formulations, including green tea extract and tocopherol [18,19], making attribution of radical-scavenging activity to the polynucleotide itself difficult. The current manufacturing process is excipient-free at the final purified-product stage. The loss of DPPH activity across purification therefore supports removal of co-extracted, non-polynucleotide constituents capable of hydrogen-atom transfer rather than enrichment of a conventional broad-spectrum antioxidant. The reductions in protein nitration observed in cells and lungs therefore occurred with a material of weak conventional antioxidant capacity and are more readily attributed to biological actions of the polynucleotide than to direct radical quenching by co-extracted antioxidants.

The contrast between assays is compatible with differences in reaction mechanism and solvent accessibility, as ABTS and DPPH responses can depend on both electron- and hydrogen-transfer chemistry as well as solvent conditions [32]. Limited solubility of the polynucleotide in the alcoholic DPPH system may also contribute; this possibility was not directly tested here.

That salmon-derived PDRN occupies essentially the same position in the assay plane (Figure 2c) indicates that this profile is a property of purified polynucleotide preparations generally rather than of the algal source. For the intended application—replacing an animal-derived material—this comparability is the relevant result.

### 3.3. Treatment Schedule Differs Between Systems

The in vitro experiments used co-treatment, whereas earlier reports employed 12 h pre-treatment and the present animal experiments used five days of oral pre-treatment. This distinction is mechanistically relevant. A_2A_ receptors are Gs-coupled and commonly signal through the adenylyl cyclase–cAMP–PKA axis, with documented modulation of MAPK pathways [33]. Treatment timing may therefore influence whether receptor-proximal signalling precedes or accompanies LPS-induced transcriptional priming.

**Figure 6:**
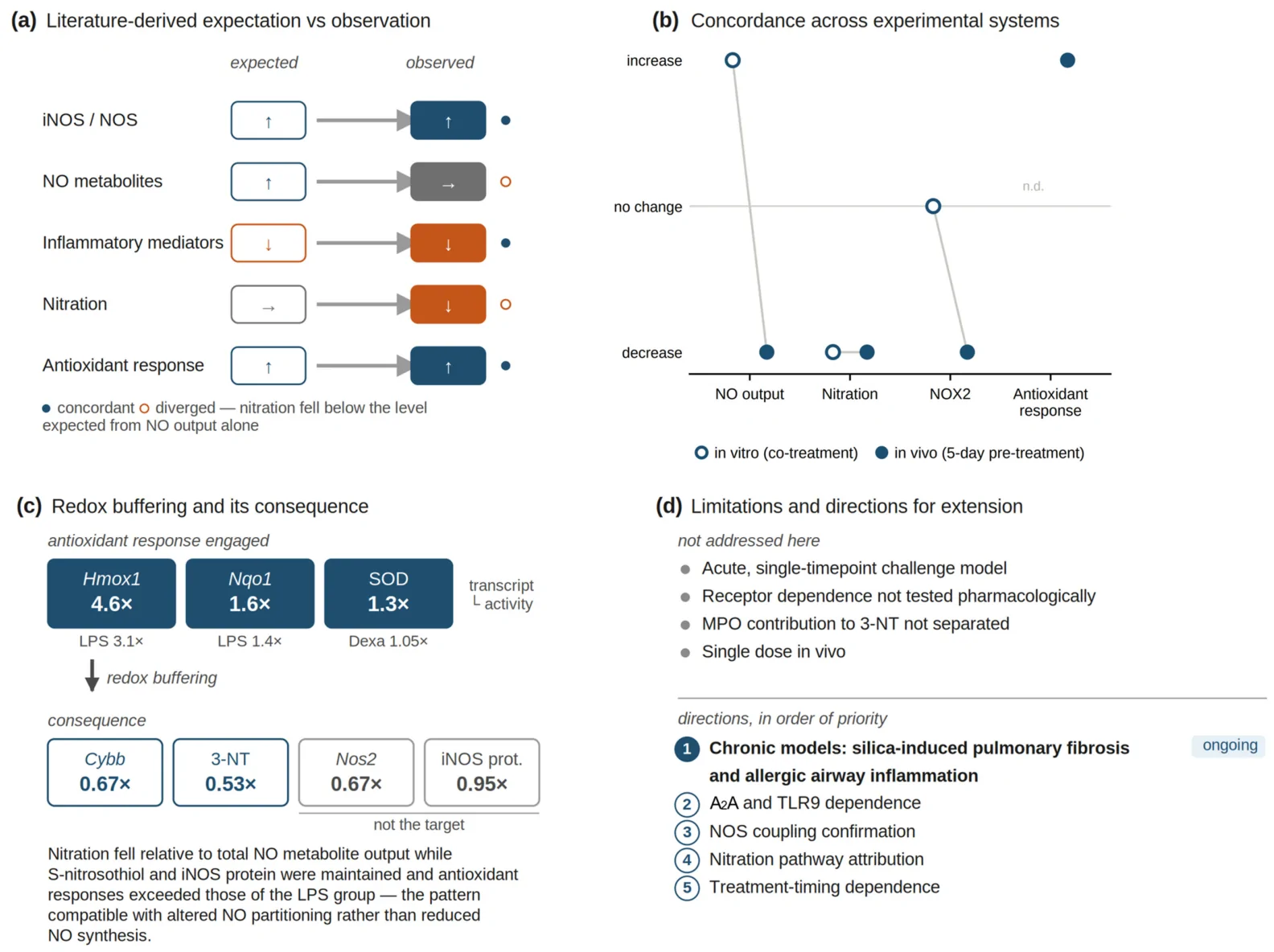
Convergence of literature-derived expectations with the present observations, and directions for extension. Throughout the figure, blue denotes an increase, orange a decrease, and grey no change relative to the LPS group. (**a**) Directions expected from the survey in Figure 4 alongside those observed here; the grey arrows lead from the expected to the observed direction, and the filled and open circles indicate concordance and divergence, respectively. (**b**) Directions observed in the two experimental systems, which differed in treatment schedule; n.d., not determined. (**c**) Antioxidant response markers and the accompanying changes in *Cybb*, 3-nitrotyrosine, *Nos2*, and iNOS protein, expressed relative to the LPS group. the values beneath the upper boxes give the corresponding change in the comparator named, and the downward arrow links the antioxidant response to its consequence. (**d**) Questions not addressed by the present design and directions for extension in order of priority.

Consistent with this, the two systems in the present study differed in NO output—increased nitrite in vitro, and reduced total NO metabolites in lung tissue—but agreed on the direction of nitration, which fell in both (Figure 6b).

Reduced ERK and p38 phosphorylation has been reported 10–15 min after LPS stimulation for a related preparation [18], while nitrite was measured at 24 h. The MAPK observation is robust as a description of early signalling, but the interval between the two endpoints means that a causal link between MAPK inhibition and altered nitrite accumulation is not established by that design. A_2A_–cAMP–PKA signalling can itself modulate MAPK activation [33], so reduced phosphorylation may represent a consequence of receptor-proximal events rather than the proximate cause.

### 3.4. Two Interpretations of the Observed Profile

The profile reported here admits two interpretations. Under the first, PS-PDRN acts primarily as an anti-inflammatory agent and the reduction in nitrative markers follows from diminished inflammatory signalling. Under the second, PS-PDRN acts on the redox environment in which iNOS-derived NO is partitioned, lowering the fraction of NO diverted toward nitration.

The present data do not separate these definitively. Several observations, however, are more readily accommodated by the second. *Nos2* remained at 67% of the LPS group and lung iNOS protein at 95%, while 3-NT fell to 53%; dexamethasone produced the converse (44%, 48%, and 66%). iNOS protein abundance is an indirect index of enzyme activity, so the comparison is more informative when nitration is expressed relative to total NO metabolite output, the integrated product of NO synthesis. On that basis, lung 3-nitrotyrosine per unit of NO metabolites was 0.87 times the LPS value under PS-PDRN and 1.89 times under dexamethasone, and in macrophages 3-nitrotyrosine fell to 77% while total NO metabolites were 114% of the LPS group. The two treatments therefore moved nitration in opposite directions relative to NO output. This pattern is compatible with a difference in NO partitioning. Total NO metabolites integrate synthesis over the sampling interval and do not resolve local enzyme activity, NOS uncoupling, or myeloperoxidase-dependent nitration, and functional evidence will be required to distinguish these alternatives.

*Hmox1* and *Nqo1* were induced above the LPS group, and total SOD activity was likewise higher, indicating that antioxidant responses were engaged rather than suppressed (Figure 6c). The same direction was observed in vitro, where *Nqo1* transcript was higher under PS-PDRN than under LPS alone (Figure 3j). Dexamethasone lowered *Hmox1* to 42% and left SOD activity unchanged relative to LPS. The difference in nitration between the two treatments is therefore not attributable to greater superoxide-removal capacity in the dexamethasone group.

MPO activity was reduced less by PS-PDRN than by dexamethasone (57% versus 29%), as were body temperature and pulmonary edema. Greater suppression of the measured inflammatory endpoints by dexamethasone was accompanied by a smaller reduction in 3-nitrotyrosine than with PS-PDRN. However, this difference alone does not establish altered NO partitioning or exclude contributions from anti-inflammatory effects.

We present this study as an early-phase scoping analysis consistent with, but not establishing, a redox-partitioning mechanism.

### 3.5. Relation to A_2A_ Signalling and to the Redox-Buffered Model

Salmon-derived PDRN acts as an adenosine A_2A_ receptor agonist, and A_2A_ activation reduces cytokine production in collagen-induced arthritis and spinal cord injury models [34,35]. In testicular ischemia–reperfusion, the protective effect of PDRN is abolished by the A_2A_ antagonist DMPX [23]. As PS-PDRN is a polynucleotide of comparable chemical class and closely similar radical-scavenging profile, A_2A_ engagement is a plausible contributor. Receptor dependence was not tested here, and this remains the most direct outstanding question (Section 3.8). PS-PDRN is a preparation of short DNA fragments, which places endosomal TLR9, the receptor for double-stranded DNA, alongside A_2A_ as a candidate input. TLR9 engagement signals through NF-κB and would account for the maintained iNOS expression and the low level of nitrite production observed without LPS (Figure S4b), and the reduction in protein nitration relative to NO output would then arise downstream of NO synthesis rather than at the level of receptor engagement. The two candidates are separable pharmacologically, by A_2A_ antagonism and by inhibitory oligodeoxynucleotides or chloroquine for TLR9.

**Figure 7:**
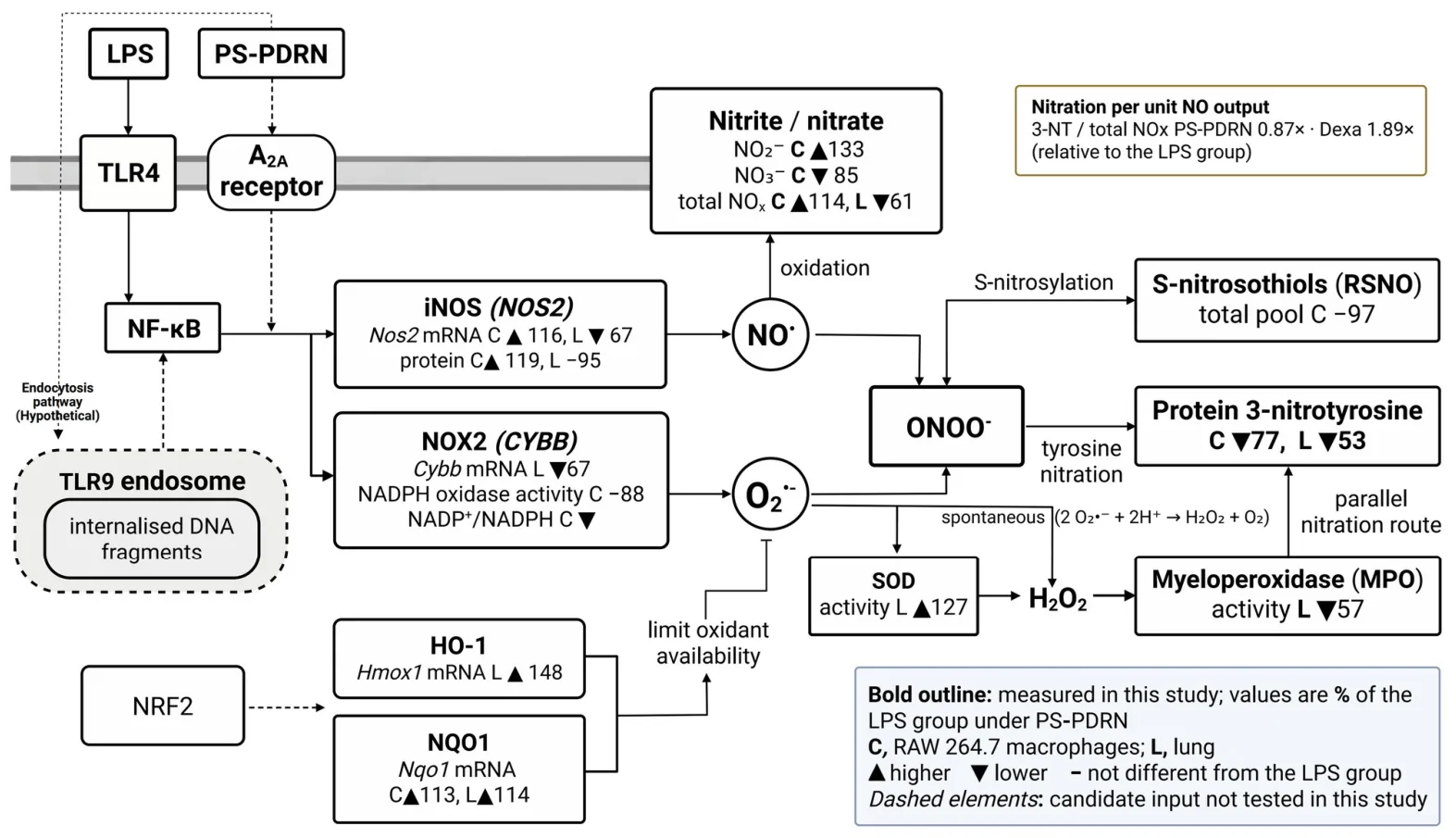
Reactive nitrogen species pathways examined in this study. LPS engagement of TLR4 induces iNOS (NOS2) and NADPH oxidase 2 (NOX2/CYBB) through NF-κB. iNOS-derived NO is oxidized to nitrite and nitrate, transferred to protein thiols as S-nitrosothiols, or combines with NOX2-derived superoxide to form peroxynitrite, which nitrates protein tyrosine residues to 3-nitrotyrosine. Superoxide is also converted to hydrogen peroxide by superoxide dismutase and by spontaneous dismutation, providing the substrate for myeloperoxidase-dependent nitration as a parallel route to 3-nitrotyrosine. The Nrf2 targets HO-1 (*Hmox1*) and NQO1 (*Nqo1*) limit oxidant availability. Adenosine A_2A_ receptor signalling and endosomal TLR9, the receptor for internalized DNA fragments, are shown as candidate upstream inputs for PS-PDRN that were not tested here. Nodes with a bold outline were measured in this study, with values given as a percentage of the LPS group under PS-PDRN in RAW 264.7 macrophages (C) and lung (L). The box at the upper right gives lung 3-nitrotyrosine expressed per unit of total NO metabolites, as a fraction of the same ratio in the LPS group.

More broadly, the observed pattern is consistent with a redox-buffered model of iNOS-derived NO signalling, in which sustained NO production is compatible with restrained inflammatory output when superoxide competition is limited [12]—a pattern documented in endotoxin-tolerant macrophages and in models where iNOS deletion exacerbates rather than attenuates disease [6,7,25]. One element of that model is directly supported here: the S-nitrosothiol pool was maintained under PS-PDRN while nitration fell (Figure 3g), the partitioning between S-nitrosylation and nitration that the model requires. The framework further predicts attenuation of inflammasome priming under such conditions; these endpoints were not measured here and represent a defined target for subsequent testing. Figure 7 places the readouts measured in this study within the intracellular reactive nitrogen species network.

### 3.6. Material Differentiation and Translational Relevance

Source-genome context is relevant to the intended use of *Porphyra*/*Pyropia* sp. as a non-animal PDRN source. The reference nuclear genomes of *P. yezoensis* and *O. keta* differ substantially in GC composition, yet the purified algal and salmon-derived polynucleotide preparations converged closely in the DPPH/ABTS assay plane (Figure 2c). The combination of cultivated marine biomass, excipient-free purification, a defined low-molecular-weight fragment profile, and functional comparability with salmon-derived PDRN supports PS-PDRN as a candidate PDRN-class material platform. Potential advantages in supply resilience, cost of goods, sustainability, and regulatory standardization remain translational hypotheses and require dedicated scale-up, techno-economic, life-cycle, and batch-to-batch studies.

### 3.7. Limitations

Receptor dependence was not tested pharmacologically; A_2A_ antagonism and TLR9 blockade will be required to establish which receptor, if either, mediates the observed effects. MPO activity was reduced to an extent comparable with the reduction in 3-nitrotyrosine, and 3-nitrotyrosine is not an exclusive footprint of peroxynitrite [36]; the relative contributions of peroxynitrite- and myeloperoxidase-dependent nitration were therefore not separated. S-nitrosothiol was quantified as a total ascorbate-dependent pool without identification of the S-nitrosylated target proteins, and iNOS protein was measured by immunoassay rather than by immunoblot, so neither enzyme dimerization state nor the identity of the modified proteins was resolved. NOS catalytic activity was not measured directly; total NO metabolite output served as its integrated index and does not resolve local or time-dependent differences in enzyme activity. Total SOD activity was measured without resolving isoforms, so mitochondrial and extracellular contributions could not be distinguished. Nrf2 engagement was inferred from target-gene expression and SOD activity; nuclear translocation and DNA-binding activity of Nrf2 itself were not measured. The approximately 16% reduction in WST-8 signal under PS-PDRN at 50 µg/mL relative to LPS alone may have indirectly influenced the cellular readouts. A single time point was examined, so the timing dependence proposed in Section 3.3 remains a hypothesis. In vivo experiments used a single dose, selected with reference to dose ranges established for related preparations. Because antioxidant response gene expression was assessed only at the endpoint after LPS challenge, it could not be determined whether induction occurred during the pre-treatment period or in response to the challenge itself; a PS-PDRN-only group without LPS challenge would be required to distinguish these in vivo; chronic models now in progress, in silica-induced pulmonary fibrosis and in allergic airway inflammation, include such a group. The present model is an acute LPS challenge; extrapolation to chronic airway inflammation, including chronic obstructive pulmonary disease, requires validation in chronic models. A chronic airway inflammation model is currently underway.

### 3.8. Directions for Extension

Direction for extension include, in order of priority (Figure 6d): validation in a chronic airway inflammation model, currently underway; pharmacological testing of A_2A_ receptor dependence; confirmation of NOS coupling status by BH4/BH2 quantification; attribution of nitration to peroxynitrite- versus myeloperoxidase-dependent routes; and examination of treatment-timing dependence across pre-treatment and co-treatment schedules. Oxidized protein products have separately been implicated in the regulation of NOS activity in the lung, and this axis represents an additional candidate route for the attribution of nitration [37].

## 4. Materials and Methods

### 4.1. Preparation and Characterization of PS-PDRN

PS-PDRN (AIO Marine Plant PDRN) and the corresponding pre-purification intermediate were supplied by All In One GENETECH Ltd. (Busan, Republic of Korea). The source material was cultivated *Porphyra*/*Pyropia* sp. obtained from Wando, Jeollanam-do, Republic of Korea. PS-PDRN was produced using an updated excipient-free purification workflow associated with Korean Patent Nos. 10-2249241 and 10-2584027. The process comprised raw-material preparation, protein removal and degradation, removal of residual impurities and purification, washing and drying, and controlled molecular-weight reduction (Figure 1b). The pre-purification intermediate was used as a process control. Commercial salmon-derived PDRN (MedChemExpress, Monmouth Junction, NJ, USA) was included as a reference material for radical-scavenging characterization. Catalogue and lot numbers of the commercial assay kits and key reagents are listed in Table S2.

Low-molecular-weight character was assessed by electrophoresis on a 1.5% agarose gel in 1× TAE buffer for 60 min against a 25/100 bp Mixed DNA Ladder (D-1020; Bioneer, Daejeon, Republic of Korea), performed by the manufacturer on the lot used in this study (Figure 1a; uncropped gel in Figure S3). Purity of PS-PDRN and the salmon-derived reference was assessed by UV spectrophotometry. Absorbance at 230, 260, and 280 nm was measured in UV-transparent 96-well plates (Corning 3635, Corning, NY, USA) on a PowerWave XS2 reader (BioTek Instruments, Winooski, VT, USA), with readings normalized to a 10 mm pathlength; DNA concentration was calculated from A_260_, and the A_260_/A_280_ and A_260_/A_230_ ratios served as indices of protein and of carbohydrate or organic contaminants, respectively. Residual protein was quantified by BCA assay against a bovine serum albumin standard (562 nm), and residual polysaccharide by the phenol–sulfuric acid method against a glucose standard (490 nm); both were expressed as a percentage of DNA content. Each measurement was performed in triplicate. Total polyphenol content was determined by the Folin–Ciocalteu method. The manufacturer’s quality-control value cited for the A_260_/A_280_ ratio was obtained by NanoDrop spectrophotometry (NANO-400A; Allsheng, Hangzhou, China). Endotoxin was quantified using the Pierce Rapid Gel Clot Endotoxin Assay Kit (Thermo Fisher Scientific, Waltham, MA, USA).

For source-genome context, the *Pyropia yezoensis* strain U-51 reference nuclear assembly (GCA_054658335.1; urPyrYezo_2.1; 104,073,448 bp; 227 contigs; GC 65.08%) was obtained from NCBI. Two further Bangiales reference nuclear assemblies were obtained from NCBI for taxonomic context: *Pyropia haitanensis* isolate PH40 (GCA_008729055.1; OUC_PyHait; 53,254,617 bp; 194 scaffolds; GC 71.26%) and *Porphyra umbilicalis* isolate 4086291 (GCA_002049455.2; P_umbilicalis_v1; 87,698,808 bp; 2125 scaffolds; GC 65.78%), the latter after exclusion of the plastid genome so that all three assemblies were compared on a nuclear-only basis. GC contents were calculated from assembly base counts. For each assembly, circular visualization displayed the contigs or scaffolds sequentially, ordered by decreasing length, together with GC content in 50 kb windows, the density of short (101 bp) windows with GC below 50%, and soft-masked repeat density; the circular arrangement is a visualization convention and does not imply a circular nuclear genome. The *Oncorhynchus keta* reference nuclear assembly (GCA_023373465.1; Oket_V2; GC 43.89%) was included solely as a source-level comparator for a conventional salmon PDRN source and does not represent sequencing of the commercial salmon-derived PDRN preparation used in the radical-scavenging assays. The deoxynucleotide mole fractions shown in Figure 1c were derived from the assembly GC content assuming Chargaff base pairing.

### 4.2. Radical-Scavenging Assays

DPPH and ABTS radical-scavenging activities were determined in parallel at 15.6, 31.3, 62.5, 125, and 250 µg/mL, with butylated hydroxytoluene as the positive control. Scavenging activity was expressed as percentage reduction relative to the corresponding control. The ABTS-to-DPPH selectivity index was calculated at 250 µg/mL. Assays were performed in three independent experiments.

### 4.3. Cell Culture and Treatment

RAW 264.7 murine macrophages (ATCC, Manassas, VA, USA) were maintained in DMEM supplemented with 10% FBS and 1% penicillin-streptomycin at 37 °C under 5% CO_2_. PS-PDRN (25 or 50 µg/mL) and LPS (200 ng/mL; *Escherichia coli* O55:B5, Sigma-Aldrich, St. Louis, MO, USA) were added simultaneously, and cells were harvested after 24 h; no pre-treatment interval was used. APX-115 (5 µM; Cayman Chemical, Ann Arbor, MI, USA) and 1400W (10 µM; Sigma-Aldrich) were used as pharmacological comparators on the same co-treatment schedule. Cell viability was determined by WST-8 assay (Biomax, Seoul, Republic of Korea) after 24 h exposure to PS-PDRN (12.5–100 µg/mL), APX-115, or 1400W, each with or without LPS, and viability was expressed relative to untreated cells or, for LPS-treated wells, to the LPS group (Figure S4). Viability was interpreted relative to the specified reference group. The 70% threshold described in ISO 10993-5 [38] was used only as contextual guidance, because normalization to the LPS group does not establish the absence of cytotoxicity relative to untreated cells.

### 4.4. NO Metabolites, Protein Nitration, S-Nitrosothiol, and iNOS Protein

Nitrite and nitrate were determined using the EZ-Nitric Oxide Assay Kit (DoGenBio, Seoul, Republic of Korea). Nitrate was measured after vanadium(III) chloride reduction, and reduction efficiency was determined and used for correction. Nitrite and nitrate were reported separately, and the NO_2_^−^/NO_3_^−^ ratio was calculated for each replicate. 3-Nitrotyrosine was quantified by ELISA (Elabscience, Wuhan, China) according to the manufacturer’s instructions and normalized to total protein determined by BCA assay. Total S-nitrosothiol was measured with a biotin-switch assay kit (ab236207, Abcam, Cambridge, UK) according to the manufacturer’s instructions; for every sample a parallel reaction in which ascorbate was omitted from the reduction step was carried through the identical procedure as an assay-specificity control, and values were expressed relative to untreated control samples. iNOS protein was quantified with a mouse NOS2/iNOS ELISA kit (E-EL-M0696, Elabscience) in cell lysates and lung homogenates, normalized to total protein determined by BCA assay, and expressed relative to untreated control samples.

### 4.5. NADPH Oxidase Activity and NADP^+^/NADPH

NADPH oxidase activity was measured using a colorimetric NADPH oxidase assay (Elabscience) by monitoring NADPH consumption at 340 nm. Initial linear rates were used, the APX-115-sensitive fraction was taken as NADPH oxidase activity, and values were normalized to protein content. NADP^+^ and NADPH were measured using a colorimetric assay (Elabscience), and the NADP^+^/NADPH ratio was calculated for each replicate.

### 4.6. Animals and Induction of Acute Lung Injury

Six-week-old male BALB/c mice (18–21.5 g) were purchased from Raon Bio (Yongin, Republic of Korea) and acclimatized for more than one week at 23 ± 2 °C and 40–60% relative humidity under a 14 h light/10 h dark cycle, with free access to standard chow and water. Animals were pooled and then allocated at random to four groups (*n* = 8 per group): control, LPS, LPS + dexamethasone, and LPS + PS-PDRN. The model was adapted from an intranasal LPS-induced acute lung injury protocol previously established in this laboratory [15]. All procedures were approved by the Institutional Animal Care and Use Committee of Konyang University (P-25-19-A-01; approved 16 October 2025).

PS-PDRN (100 µg/mouse) and dexamethasone (1 mg/kg) were administered by oral gavage once daily for five consecutive days (days 0–4). The control and LPS groups received an equal volume of distilled water on the same schedule. Acute lung injury was induced on day 4, after the final treatment, by intranasal administration of LPS (*Escherichia coli* O55:B5; 1 µg/µL, 50 µL/mouse). Mice were anesthetized by inhalation, with induction at 3–4% isoflurane in a chamber and maintenance at 1.5–2% isoflurane in oxygen at 1 L/min. Once loss of the righting reflex was confirmed, each mouse was held vertically and the LPS solution was instilled slowly into both nostrils with a micropipette, allowing it to be drawn into the lungs during inhalation. BALF, lung tissue, and blood were collected 12 h after challenge. The PS-PDRN dose was selected with reference to the range previously evaluated for related *Pyropia*-derived polynucleotide preparations in the same model [19]. Body temperature was measured rectally before euthanasia. Mice were euthanized 12 h after challenge by inhalation of an overdose of isoflurane, followed by exsanguination via cardiac puncture. For assessment of pulmonary edema, lungs were weighed before and after drying at 60 °C for 48 h, and the wet/dry ratio was calculated.

### 4.7. Lung Tissue and BALF Analysis

For BALF collection, a 22-gauge catheter was inserted into the exposed trachea and the lungs were lavaged three times with 0.9 mL of ice-cold PBS. Total BALF NO metabolites were calculated as the sum of nitrite and nitrate. BALF and lung tissue were treated as separate biological compartments and were not combined for analysis.

Lung tissue was homogenized in RIPA buffer containing protease and phosphatase inhibitors and clarified by centrifugation. Protein concentration was determined by BCA assay. Nitrite, nitrate, and 3-nitrotyrosine were measured as described above. MPO activity was measured using a myeloperoxidase activity assay (Elabscience), and total SOD activity using the EZ-Superoxide Dismutase Assay Kit (DoGenBio).

Serum TNF-α, IL-1β, and CCL5 were quantified by ELISA (Invitrogen, Carlsbad, CA, USA) according to the manufacturer’s instructions (Figure S2).

### 4.8. Quantitative Real-Time PCR

Total RNA was extracted from lung tissue using TRIzol reagent (iNtRON Biotechnology, Seongnam, Republic of Korea), and cDNA was synthesized using a cDNA synthesis kit (GenDEPOT, Katy, TX, USA). Quantitative real-time PCR was performed using a CFX96 system (Bio-Rad, Hercules, CA, USA). Validated mouse primer sets from OriGene Technologies (Rockville, MD, USA) were used for *Nos2*, *Cybb*, *Hmox1*, *Nqo1*, *Gapdh*, and *Actb*; accession and catalogue numbers are listed in Table 2. Relative expression was calculated using the 2^−ΔΔCt^ method [39], normalized to the geometric mean of *Gapdh* and *Actb*, and expressed relative to the control. The same procedure and the same *Nos2* and *Nqo1* primer sets were applied to RAW 264.7 lysates for the in vitro transcript measurements.

**Table 2:** Primer pairs used for quantitative real-time PCR.

Gene	Accession No.	Catalogue No.
*Nos2*	NM_010927	MP208933
*Cybb*	NM_007807	MP203212
*Hmox1*	NM_010442	MP206003
*Nqo1*	NM_008706	MP208646
*Gapdh*	NM_008084	MP205604
*Actb*	NM_007393	MP200232

### 4.9. Structured Literature Survey

A structured PubMed survey was conducted for primary experimental studies published during the preceding 30 years (1996–2026) that reported NOS/iNOS or NO status together with at least one inflammatory, nitrative, or tissue-outcome measure in the same model. Searches used combinations of terms related to iNOS, nitric oxide, inflammation, nitration, and PDRN, supplemented by reference-list screening. Reviews and hypothesis articles were excluded from directional coding and used only for contextual discussion. The survey was descriptive rather than systematic; no protocol was registered and no formal risk-of-bias assessment was performed.

### 4.10. Statistical Analysis

Statistical analyses were performed using SAS 9.4 (SAS Institute Inc., Cary, NC, USA). Data are presented as mean ± SD, with *n* = 3 independent experiments for in vitro measurements (three separate experiments, each conducted on an independent plate, for the viability assay) and *n* = 8 animals per group for in vivo measurements; individual values are shown in the figures. Comparisons between the control and LPS groups were evaluated using Welch’s *t*-test, which does not assume equal variances. Treatment effects were evaluated by one-way ANOVA followed by Dunnett’s test against the LPS group; in the radical-scavenging assays each preparation was compared with the concentration-matched BHT reference, and in the viability assay each condition was compared with its corresponding untreated or LPS-only reference. Calculated concentrations below zero were set to zero before further calculations. Normality of ANOVA residuals was assessed by the Shapiro–Wilk test and homogeneity of variance by the Brown–Forsythe test. Most panels met both assumptions and were analyzed as described above; for the panels in which variances were heterogeneous, which occurred among the in vitro measurements at *n* = 3, Welch’s ANOVA with the Games–Howell post hoc test was used instead. For the serum cytokine measurements in Figure S2, individual-animal values below the assay detection limit (7.8 pg/mL for IL-1β and 15.6 pg/mL for TNF-α and CCL5) were assigned one-half of the corresponding limit and retained in the analysis. Ratio variables were calculated for each replicate before group averaging.

## 5. Conclusions

An excipient-free polynucleotide purified from cultivated *Porphyra*/*Pyropia* sp. was defined at the manufacturing, electrophoretic, radical-scavenging, cellular, and in vivo levels. PS-PDRN shared the selective ABTS-over-DPPH scavenging profile of salmon-derived PDRN despite the marked difference in GC composition between the *P. yezoensis* and *O. keta* reference genomes. In inflammatory models, the material lowered protein nitration relative to total NO metabolite output while iNOS protein was maintained, and it induced antioxidant response markers without direct inhibition of NADPH oxidase in macrophages. Together, these observations characterize PS-PDRN as a process-defined, low-molecular-weight, non-animal PDRN-class marine material and provide a rationale for receptor-dependence, NOS-coupling, chronic-disease, scale-up, and industrial-standardization studies.

## Figures and Tables

**Table 1:** Comparison of the present study with earlier studies of *Porphyra*/*Pyropia*-derived polynucleotide preparations.

Feature	Kim et al., 2023 [18]	Lee et al., 2026 [19]	Present Study
Material	Ps-PDRN; extraction formulation containing green tea extract and tocopherol	Ps-PDRN; lysis formulation containing green tea extract and tocopherol; 5–1000 kDa	PS-PDRN; excipient-free at the purified-product stage; residual polyphenol 1.0 mg GAE/g
Comparators	Commercial salmon-derived PDRN	Dexamethasone	Salmon-derived PDRN and pre-purification intermediate (radical scavenging); APX-115 and 1400W (in vitro); dexamethasone (in vivo)
Models	RAW 264.7 + *E. coli* LPS; human dermal fibroblasts	Male BALB/c mice, intranasal LPS acute lung injury; RAW 264.7 + LPS	RAW 264.7 + *E. coli* O55:B5 LPS; male BALB/c mice, intranasal LPS acute lung injury
Treatment schedule	12 h pre-treatment	Intranasal (25 or 50 µg/mouse) or oral (100 or 200 µg/mouse) once daily for 3 days before LPS; 12 h pre-treatment in vitro	In vitro co-treatment, 24 h; in vivo oral pre-treatment for 5 days, 100 µg/mouse
NO-related readouts	Nitrite (Griess); iNOS protein	Not measured	Nitrite, nitrate, NO_2_^−^/NO_3_^−^, total S-nitrosothiol, 3-nitrotyrosine, iNOS protein, *Nos2*
Other readouts	p38 and ERK phosphorylation; viability; fibroblast proliferation and collagen production	Cytokines and chemokines in BALF and serum; lung cytokine mRNA; histology; BALF macrophage morphology	NADPH oxidase activity, NADP^+^/NADPH, *Cybb*, *Hmox1*, *Nqo1*, SOD, MPO, serum cytokines
Principal findings	NO production ↓, iNOS protein ↓, p38/ERK phosphorylation ↓; no cytotoxicity	Fever, pulmonary edema, cytokines, and chemokines ↓; alveolar architecture preserved	In vitro: nitrite ↑, nitrate ↓, 3-nitrotyrosine ↓, S-nitrosothiol and iNOS protein maintained; in vivo: 3-nitrotyrosine ↓ relative to total NO metabolites; *Hmox1*, *Nqo1*, and SOD ↑

↑, increased; ↓, decreased, relative to the LPS-stimulated or LPS-challenged control in each study.

## Data Availability

The original contributions presented in this study are included in the article and Supplementary Materials. The reference genome assemblies used for the source-genome analyses are publicly available from NCBI under accession numbers GCA_054658335.1, GCA_008729055.1, GCA_002049455.2, and GCA_023373465.1. Additional data supporting the findings of this study are available from the corresponding author upon reasonable request.

## Supplementary Materials

The following supporting information can be downloaded at https://www.mdpi.com/article/10.3390/md24100361/s1: Figure S1: Model validation (body temperature and lung wet/dry ratio); Figure S2: Serum TNF-α, IL-1β, and CCL5; Figure S3: Uncropped gel image for Figure 1a; Figure S4: Effects of PS-PDRN on RAW 264.7 macrophages in the absence and presence of LPS (viability, nitrite, and nitrate); Table S1: Reported iNOS/NO responses and concurrent inflammatory, nitrative, and outcome measures; Table S2: Commercial assay kits, key reagents, and reference materials used in this study. References [26,27,29,30,40] are cited in Table S1.
