# Supplementary material for: An Excipient-Free Porphyra/Pyropia sp.-Derived Polynucleotide Modulates Nitrative Stress in LPS-Stimulated Macrophages and Murine Acute Lung Injury
Source: Mar Drugs. 2026 Oct 18;24(10):361. doi: 10.3390/md24100361 (PMC13706567; doi:10.3390/md24100361)
Supplement: Supplementary file 1 [file marinedrugs-24-00361-s001.zip › marinedrugs-4555928-supplementary.pdf]

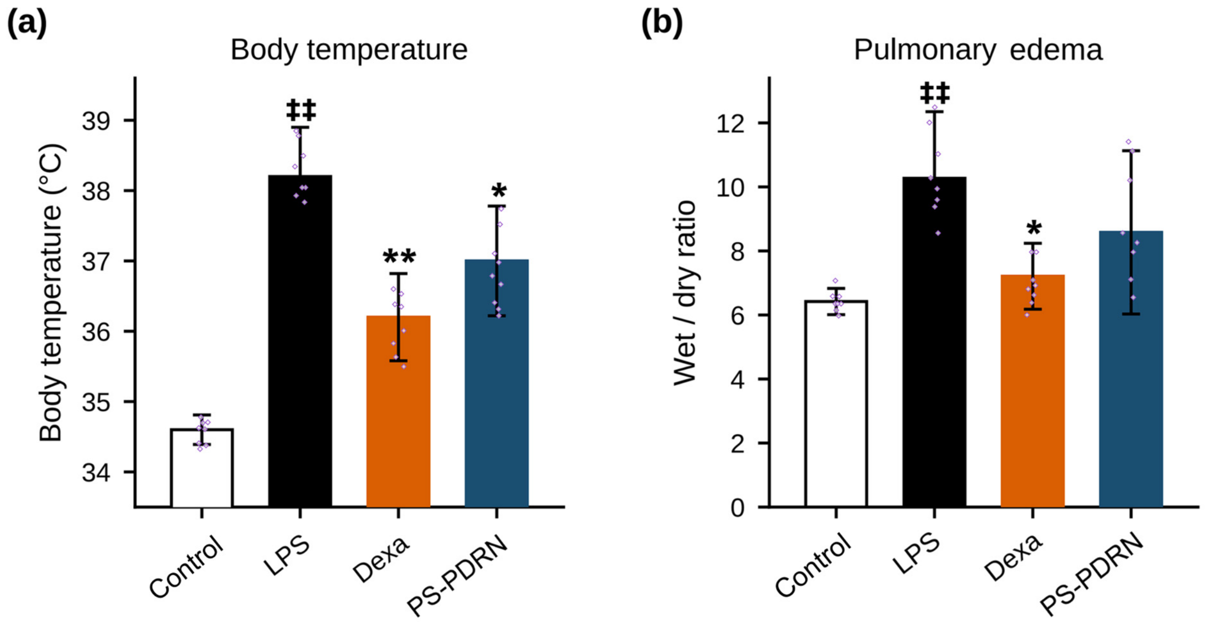

Figure S1. Model validation. (**a**) Body temperature and (**b**) lung wet/dry ratio. Data are mean ± SD of n = 8 animals per group, with individual animals shown as points. ‡‡, p < 0.01 versus the control group; *, p < 0.05; **, p < 0.01 versus the LPS group.

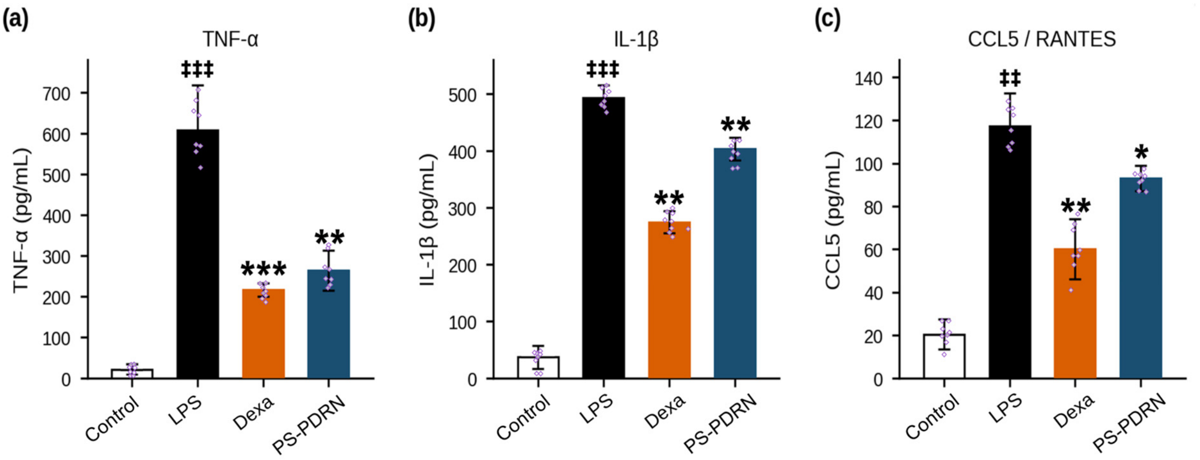

Figure S2. Serum TNF-α, IL-1β, and CCL5. Data are mean ± SD of n = 8 animals per group, with individual animals shown as points. For these serum cytokine measurements only, individual-animal values below the assay detection limit (7.8 pg/mL for IL-1β, 15.6 pg/mL for TNF-α and CCL5) were assigned one-half of the corresponding limit. ‡‡, p < 0.01; ‡‡‡, p < 0.001 versus the control group; *, p < 0.05; **, p < 0.01; ***, p < 0.001 versus the LPS group.

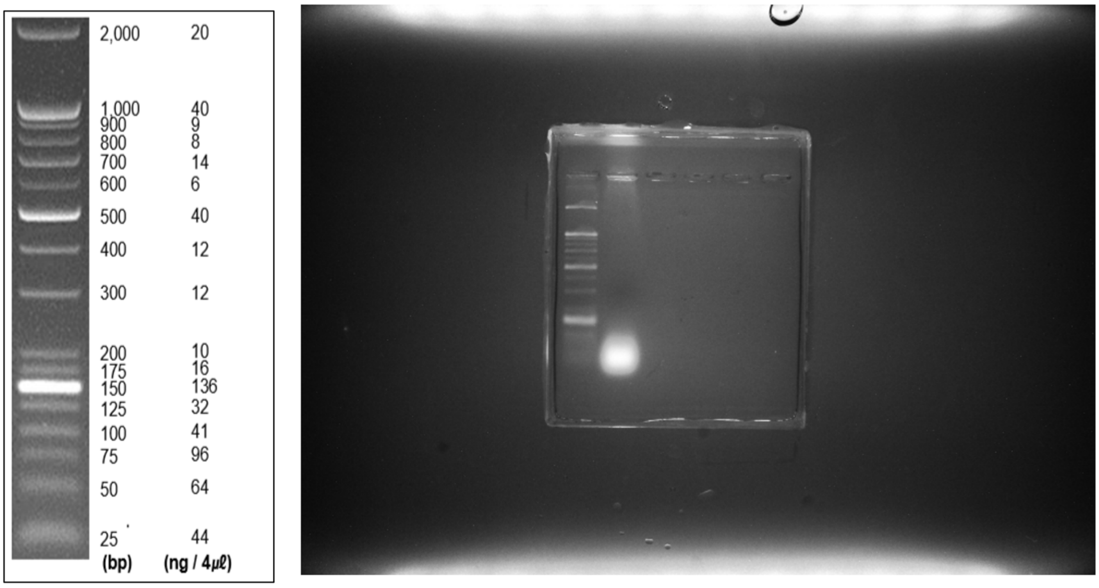

Conditions	Buffer	X1 TAE buffer
	Gel	1.5% Agarose gel
	Ladder	25/100bp Ladder(Bioneer, Korea)
	Running	60 min running

Figure S3. Uncropped gel image for Figure 1A. PS-PDRN was resolved on a 1.5% agarose gel in 1× TAE buffer for 60 min against a 25/100 bp Mixed DNA Ladder (D-1020; Bioneer, Daejeon, Republic of Korea); the electrophoresis was performed by the manufacturer on the lot used in this study. The ladder specification sheet is shown at the left, and the full gel with all lanes and the ladder at the right. Brightness and contrast were adjusted uniformly across the whole image.

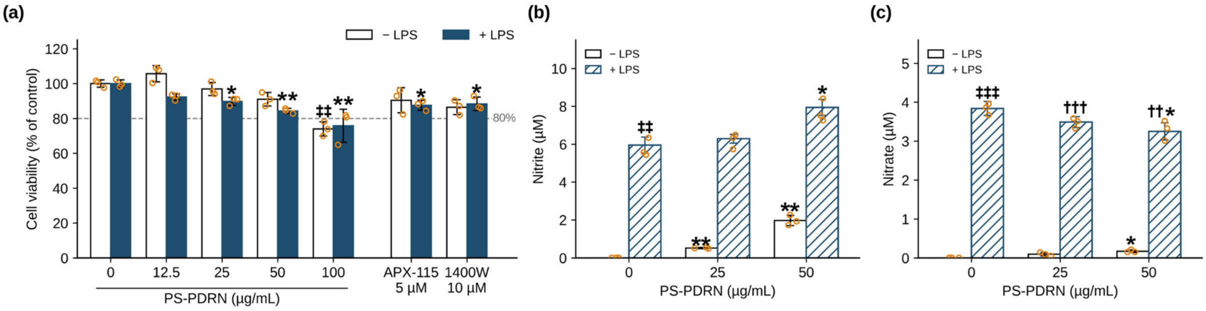

Figure S4. Effects of PS-PDRN on RAW 264.7 macrophages in the absence and presence of LPS. (**a**) Viability after 24 h exposure to PS-PDRN, APX-115 (5 µM), or 1400W (10 µM), determined by WST-8 reduction; values are expressed relative to untreated cells (− LPS) or to the LPS group (+ LPS), and the dashed line marks 80% of the corresponding reference value as a visual guide. LPS alone reduced the WST-8 signal to 78.6% of untreated cells. At 50 µg/mL, PS-PDRN with LPS gave 84.3% of the LPS-only signal; the approximately 16% reduction was considered when interpreting the functional results. (**b**) Nitrite and (**c**) nitrate concentrations

in cultures treated without LPS; hatched bars show the corresponding LPS-stimulated values from Figure 3. Data are mean ± SD of three separate experiments, each conducted on an independent plate, with individual experiments shown as points. ‡, $p < 0.05$; ‡‡, $p < 0.01$; ‡‡‡, $p < 0.001$ versus the 0 μg/mL condition without LPS; *, $p < 0.05$; **, $p < 0.01$; ***, $p < 0.001$ versus the LPS group in (**a**) or versus the 0 μg/mL condition without LPS in (**b**,**c**); †, $p < 0.05$; ††, $p < 0.01$; †††, $p < 0.001$ versus the corresponding condition without LPS.

Table S1. Reported iNOS/NO responses and concurrent inflammatory, nitrative, and outcome measures

Coding convention. All directions are reported for the condition in which iNOS or NO activity is **high** — wild-type in knockout studies, vehicle in inhibitor studies, treated in supplementation studies. This normalization allows studies using opposite experimental manipulations to be compared on a common axis.

↑ increased · → unchanged · ↓ decreased · n.r. not reported

A. Genetic loss of function

Year	Study	Model / manipulation	iNOS·NO	Concurrent findings	Relevance	DOI
1998	Yamasaki et al., *J Clin Invest*	Mouse excisional skin wound; WT vs iNOS-KO; partially selective iNOS inhibitor in WT; topical adenoviral human iNOS rescue in KO	iNOS mRNA peaks day 4–6 in WT	iNOS-KO: wound closure delayed 31%; inhibitor reproduces delay; **iNOS gene transfer fully rescues**	Causal evidence that iNOS induction is not intrinsically pathological	10.1172/JCI2067
2001	Kobayashi et al., *Am J Respir Cell Mol Biol*	WT vs iNOS-KO mice, >98% O_2 for 72 h	iNOS mRNA ↑ in WT hyperoxia	iNOS-KO shows **greater** lung injury and PMN accumulation; nitrotyrosine present in **both** WT and KO	Dissociates iNOS induction from protein nitration — nitration is not simply downstream of iNOS	10.1165/ajrcmb.24.4.4218
2011	Kelleher et al., *Am J Physiol Lung Cell Mol Physiol*	Aerosolized LPS lung injury; WT vs NOS2-KO	NOS2 ↑ after LPS, then SNO-p65 ↑	NF-κB activity ↓ concurrently; in NOS2-KO, NF-κB activity persists and inflammation is prolonged	Direct evidence of an inflammation-induced NOS2 → NF-κB negative feedback loop	10.1152/ajplung.00463.2010
2011	Niedbala et al., *PNAS*	EAE; WT vs Nos2-KO	Endogenous iNOS-derived NO	Nos2-KO: EAE severity ↑, IL-17A/IL-22 ↑; NO suppresses human and mouse Th17	Protective immune-regulatory role of iNOS/NO in autoimmune inflammation	10.1073/pnas.1100667108

Year	Study	Model / manipulation	iNOS·NO	Concurrent findings	Relevance	DOI
2013	Mao et al., *Cell Res*	Macrophage NLRP3 inflammasome; LPS-induced septic shock; WT vs iNOS-deficient	Endogenous iNOS-derived NO	NO suppresses NLRP3; iNOS deficiency increases inflammasome activation and septic injury	NO limits the ROS–mitochondrial dysfunction–inflammasome amplification loop	10.1038/cr.2013.6
2015	Lu et al., *Nat Commun*	Macrophage M1 polarization; endotoxin shock	iNOS-derived NO ↑	iNOS inhibition or knockout increases M1 polarization and inflammation; NO donor suppresses it. Mechanism involves **IRF5 tyrosine nitration** as the inhibitory signal	Strong paradox evidence; also shows that tyrosine nitration is not uniformly injurious	10.1038/ncomms7676
2017	Kitano et al., *Dermatol Res Pract*	C57BL/6 WT vs iNOS-KO; 5-mm full-thickness dorsal excisional wounds; days 1–14	Genetic loss of iNOS	KO: delayed closure, granulation tissue ↓, re-epithelialization ↓, myofibroblasts ↓, macrophage infiltration ↓, TGF-β1 and collagen 1α2 ↓	Independent replication of Yamasaki 1998	10.1155/2017/2184040

B. Pharmacological inhibition or NO supplementation

Year	Study	Model / manipulation	iNOS·NO	Concurrent findings	Relevance	DOI
1998	Binion et al., *Am J Physiol*	Primary human intestinal microvascular endothelial cells; cytokine + LPS activation	iNOS/NO ↑ on activation	iNOS inhibition **increases** leukocyte adhesion and intracellular ROS	iNOS-derived NO functions as an endogenous antioxidant and anti-adhesive factor	10.1152/ajpgi.1998.275.3.G592
1999	Stallmeyer et al., *J Invest Dermatol*	Mouse excisional cutaneous wound; selective iNOS inhibitor L-NIL	Strong iNOS induction during normal repair; suppressed by L-NIL	iNOS inhibition severely impairs re-epithelialization; keratinocyte proliferation ↓	Contradicts the interpretation that inflammation-associated iNOS induction is itself injurious	10.1046/j.1523-1747.1999.00784.x
2000	Binion et al., *Free Radic Biol Med*	Normal vs IBD-derived intestinal microvascular endothelial cells	iNOS/NO **deficient** in chronic IBD-derived cells	iNOS deficiency associated with leukocyte hyperadhesion;	Loss of iNOS can worsen the inflammatory phenotype in chronic disease	10.1016/S0891-5849(00)00391-9

Year	Study	Model / manipulation	iNOS·NO	Concurrent findings	Relevance	DOI
				NO provision normalizes it		
2020	Iwata et al., *Biochem Biophys Rep*	Rat carrageenan pleurisy; local NOS inhibition or NO supplementation	Local NO availability ↑	Leukocyte infiltration ↓, vascular permeability ↓, oxidative-stress enzyme activity ↓	NO elevation and reduced oxidative stress can coexist (iNOS specificity limited)	10.1016/j.bbrep.2020.100790

C. Adaptive states

Year	Study	Model / manipulation	iNOS·NO	Concurrent findings	Relevance	DOI
1998	Jacobs et al., *Clin Exp Immunol*	J774 macrophages; LPS ± IL-10	NO ↑ with IL-10	TNF-α ↓ concurrently; improved *T. cruzi* control	Classical macrophage precedent for elevated NO within an anti-inflammatory state (NO readout rather than iNOS protein)	10.1046/j.1365-2249.1998.00637.x
2024	Chang YY, Chao YH, Jean WH, Lin TY, Lu CW, *Nitric Oxide* 147:1–5	RAW 264.7; LPS_1 100 ng/mL 24 h → LPS_2 100 ng/mL 24 h (endotoxin tolerance and recovery)	iNOS, NO, and nitrite ↑ during tolerance	TNF-α and IL-6 ↓ concurrently; on tolerance recovery the pattern reverses (iNOS/NO ↓, cytokine rebound ↑); 1400W impairs tolerance development	Closest contemporary macrophage evidence to the present observations	10.1016/j.niox.2024.03.005

D. PDRN-specific

Year	Study	Model / manipulation	iNOS·NO	Concurrent findings	Relevance	DOI
2008	Bitto et al., *Crit Care Med*	Male C57BL mice; deep-dermal second-degree thermal burn (80 °C, 10 s); PDRN 8 mg/kg/day i.p. × 14 d; sacrifice day 7/14	**iNOS** ↑, wound NO products ↑	TNF-α ↓, re-epithelialization ↑, wound closure faster, ECM maturation ↑, microvessel density ↑, VEGF ↑	Most direct PDRN precedent: iNOS/NO ↑ without worsened inflammation or outcome	10.1097/CCM.0b013e318170ab5c
2012	Polito et al., *J Vasc Surg*	Male Sprague–Dawley rat; H-shaped ischemic skin double flap; PDRN 8	Day 3 iNOS protein ↑ (vehicle	HIF-1α ↓, VEGF ↑, blood flow recovery ↑ from day 5; complete	Second direct PDRN precedent: iNOS ↑ concurrent	10.1016/j.jvs.2011.07.083

Year	Study	Model manipulation	/ iNOS·NO	Concurrent findings	Relevance	DOI
		mg/kg i.p.; days 3, 5, 10	3.9 ± 0.6 → PDRN 5.3 ± 1); nitrite measured	re-epithelialization; ; fibroblast-rich granulation tissue	with reduced hypoxic injury	
2012	Minutoli et al., *Int J Androl*	Rat testicular ischemia/reperfusion; PDRN 8 mg/kg i.p.; ± A_{2A} antagonist DMPX	eNOS mRNA ↑ at days 1, 7, 30	Edema and necrosis ↓, spermatogenic recovery ↑; **DMPX abolishes the PDRN effect**	Pharmacological evidence for A_{2A} dependence of PDRN action; NOS isoform differs from the other entries	10.1111/j.1365-2605.2011.01194.x

E. Contrasting PDRN report

Year	Study	Model manipulation	/ iNOS·NO	Concurrent findings	Relevance	DOI
2023	Kim et al., *Cell Biochem Funct*	RAW 264.7 + *E. coli* LPS; *Porphyra*-derived Ps-PDRN vs commercial PDRN; **12 h pre-treatment**; Griess assay only	**iNOS protein** ↓, **NO production** ↓ via p38/ERK suppression	Anti-inflammatory effect; no cytotoxicity	Reports a PDRN preparation that lowered iNOS and NO. Differs from the present work in preparation composition, treatment timing (pre- versus co-treatment), and NO readout (nitrite alone versus fractionated metabolites); compared with the present study in Table 2 of the main text.	10.1002/cbf.3840

F. Conceptual synthesis (not coded as primary evidence)

Year	Study	Type	Content	DOI
2026	Lee, *Med Hypotheses* 210:111942	Hypothesis article; no primary data	Proposes that sustained high-flux iNOS-derived NO partitions between S-nitrosylation and cGMP–PKG signalling under buffered redox conditions, versus peroxynitrite-mediated injury under unbuffered conditions; proposes the NOx:3-NT ratio as a partitioning readout	10.1016/j.mehy.2026.111942

Notes on interpretation

Across sections A–D, the high-iNOS condition is associated with **reduced** inflammatory output and **improved or unchanged** tissue outcome in every entry. Where nitration or ROS were measured, they were unchanged (Kobayashi 2001) or reduced (Binion 1998, Mao 2013, Iwata 2020), with the exception of Lu 2015, in which tyrosine nitration of IRF5 constitutes the inhibitory signal rather than an injury marker.

Section E is the single retrieved entry in which a PDRN preparation lowered rather than raised iNOS and NO. It differs from the present work in preparation composition, treatment schedule, and NO readout; these differences are compared in Section 3.1 and Table 2 of the main text.

Section F is listed for completeness. It contains no primary data and is therefore excluded from the directional map in Figure 4.

Table S2. Commercial assay kits, key reagents, and reference materials used in this study.

**Item**	**Supplier**	**Catalogue No.**	**Lot No.**	**Used for**
Lipopolysaccharide, *Escherichia coli* O55:B5	Sigma-Aldrich	L2880	0000135217	Cells, mice
Dexamethasone	Sigma-Aldrich	D1756	WXBD7785V	Mice
APX-115	Cayman Chemical	41110	0710678	Cells; NADPH oxidase assay
1400W	Sigma-Aldrich	W4262	0000131222	Cells
Salmon-derived PDRN (reference material)	MedChemExpress	HY-W250160	1188979	Radical scavenging
DPPH	Sigma-Aldrich	D9132	STBF5255V	Radical scavenging
ABTS	Sigma-Aldrich	A1888	061M5308V	Radical scavenging
Butylated hydroxytoluene	Sigma-Aldrich	B1378	BCBW5813	Radical scavenging
Gallic acid	Sigma-Aldrich	G7384	SLBV4566	Polyphenol content
Folin–Ciocalteu reagent	Sigma-Aldrich	F9252	BCCN2098	Polyphenol content
Pierce Rapid Gel Clot Endotoxin Assay Kit	Thermo Fisher Scientific	A43881	0AA7650900	Endotoxin
25/100 bp Mixed DNA Ladder	Bioneer	D-1020	BQ-Q8-012-04	Electrophoresis
WST-8 cell viability assay kit	Biomax	QM1000	BCQF11E06	Cell viability
EZ-Nitric Oxide Assay Kit	DoGenBio	DG-NO500	DLS2205	Nitrite, nitrate
3-Nitrotyrosine ELISA kit	Elabscience	E-EL-0040	WP08XO42H8805	3-Nitrotyrosine
Biotin Switch Assay Kit (S-nitrosothiol)	Abcam	ab236207	2032608	S-nitrosothiol
Mouse NOS2/iNOS ELISA kit	Elabscience	E-EL-M0696	WP076JT8B6248	iNOS protein
NADPH oxidase activity assay kit	Elabscience	E-BC-K815-M	WZ07H2ZFD0668	NADPH oxidase
$NADP^+$/NADPH assay kit	Elabscience	E-BC-K803-M	WK100RHXZ0572	$NADP^+$/NADPH
Myeloperoxidase activity assay kit	Elabscience	E-BC-K074-M	WZ050HDF85769	MPO
EZ-Superoxide Dismutase Assay Kit	DoGenBio	DG-SOD400	DLS2204	Total SOD

Item	Supplier	Catalogue No.	Lot No.	Used for
BCA protein assay kit	DoGenBio	DG-BCA500	DLS2205	Protein normalisation
Mouse TNF-α ELISA kit	Invitrogen	88-7324-88	348105-008	Serum
Mouse IL-1β ELISA kit	Invitrogen	88-7013A-88	416036-006	Serum
Mouse CCL5/RANTES ELISA kit	Invitrogen	88-56009-88	404916-002	Serum
cDNA synthesis kit	GenDEPOT	R5600-500	04082413	qPCR
